# Combining culture optimization and synthetic biology to improve production and detection of secondary metabolites in *Myxococcus xanthus*: application to myxoprincomide

**DOI:** 10.1128/spectrum.01740-24

**Published:** 2024-10-21

**Authors:** Mathieu Sourice, Charlotte Simmler, Marc Maresca, Béatrice Py, Corinne Aubert

**Affiliations:** 1 Laboratoire de Chimie Bactérienne, UMR7283, Centre National de la Recherche Scientifique, Aix-Marseille Université, IM2B, IMM, Marseille, France; 2 Institut Méditerranéen de Biodiversité et d’Ecologie Marine et Continentale, UMR7263, Centre National de la Recherche Scientifique, Aix-Marseille Université, Marseille, France; 3 Aix-Marseille Université, CNRS, Centrale Marseille, iSm2, Marseille, France; Georgia Institute of Technology, Atlanta, Georgia, USA

**Keywords:** myxobacteria, secondary metabolites, biosynthetic gene cluster, natural products, chassis strain, synthetic biology, bioactive compounds

## Abstract

Microbial secondary metabolites play crucial ecological roles in governing species interactions and contributing to their defense strategies. Their unique structures and potent bioactivities have been key in discovering antibiotics and anticancer drugs. Genome sequencing has undoubtedly revealed that myxobacteria constitute a huge reservoir of secondary metabolites as the well-known producers, actinomycetes. However, because most secondary metabolites are not produced in the laboratory context, the natural products from myxobacteria characterized to date represent only the tip of the iceberg. By combining the engineering of a dedicated *Myxococcus xanthus* DZ2 chassis strain with a two-step growth medium protocol, we provide a new approach called two-step Protocol for Resource Integration and Maximization–Biomolecules Overproduction and Optimal Screening Therapeutics (2PRIM-BOOST) for the production of non-ribosomal peptides synthetases (NRPS)/polyketides synthases (PKS) secondary metabolites from myxobacteria. We further show that the 2PRIM-BOOST strategy will facilitate the screening of secondary metabolites for biological activities of medical interest. As proof of concept, using a constitutive strong promoter, the myxoprincomide from *M. xanthus* DZ2 has been efficiently produced and its biosynthesis has been enhanced using the 2PRIM-BOOST approach, allowing the identification of new features of myxoprincomide. This strategy should allow the chances to produce and discover new NRPS, PKS, and mixed NRPS/PKS hybrid natural metabolites that are currently considered as cryptic and are the most represented in myxobacteria.

**IMPORTANCE:**

Microbial secondary metabolites are important in species interactions and are also a prolific source of drugs. Myxobacteria are ubiquitous soil-dwelling bacteria constituting a huge reservoir of secondary metabolites. However, because most of these molecules are not produced in the laboratory context, one can estimate that only one-tenth have been characterized to date. Here, we developed a new strategy called two-step Protocol for Resource Integration and Maximization–Biomolecules Overproduction and Optimal Screening Therapeutics (2PRIM-BOOST) that combines the engineering of a dedicated *Myxococcus xanthus* chassis strain together with growth medium optimization. By combining these strategies with the insertion of a constitutive promoter upstream the biosynthetic gene cluster (BGC), the production of myxoprincomide, a characterized low-produced secondary metabolite, was successfully and significantly increased. The 2PRIM-BOOST enriches the toolbox used to produce previously cryptic metabolites, unveil their ecological role, and provide new molecules of medical interest.

## INTRODUCTION

The myxobacteria are classified as a keystone taxa in the soil microbacterial food web and frequently determine community’s organization (1). They are ubiquitously found in soils and in some marine sources, and have been shown to exhibit a complex life cycle, which involves motility, predation, and the production of secondary metabolites (SM) (2). As opposed to primary metabolites (e.g., amino acids), these metabolites are usually considered as non-essential for the basic functioning of the cells. Nevertheless, they confer a definite advantage to the producing organisms under conditions of stresses governing interactions between organisms within ecosystems (e.g., predation, competition, nutriment starvation). Microbial SM most often harbor original properties with potent bioactivities such as antibacterial, antifungal, antiparasitic, antiviral, and anticancer, collectively inspiring researchers participating in drug discovery efforts (3). In the search for bacterial producers, genomes from the NCBI RefSeq database have been analyzed, and despite the low representation of sequences from Myxococcota phylum in the database, this phylum has been shown to account for 1,895 biosynthetic gene clusters (BGC) (4–9). Since more than two decades, new and intriguing SM from various myxobacteria have been constantly discovered, making these bacteria recognized as a significant producer of SM, alongside actinomycetes, and bacteria belonging to the genera *Bacillus* and *Pseudomonas* (4). Accordingly, BGC are detected in most myxobacteria genomes and represent almost 6% to 10% of their total genome. However, barely one-tenth of the corresponding SM are characterized to date. The vast majority of myxobacterial BGC have no sequence similarities with characterized BGC nor SM assigned (10). Thus, to date, characterized myxobacterial SM represent only the tip of the iceberg.

In that context, myxobacteria appear as a promising source of SM. Interestingly, most myxobacterial SM structures and mechanisms of action, such as the anticancer epothilones, have been shown to be different from those of other phyla (5, 8, 11–13). Also, unlike actinomycetes, metabolites produced by myxobacteria are generally non-glycosylated (8). Moreover, myxobacterial SM tend to be more target specific than SM from other organisms (14).

The vast majority (79%) of myxobacterial SM are synthesized by complex enzymatic machineries that are often non-ribosomal peptide synthetases (NRPS), polyketide synthases (PKS), and mixed NRPS-PKS hybrids (6, 10). In myxobacteria, as in other producer organisms, most BGC turn out to be non-expressed in laboratory growth conditions, making it difficult to identify their cognate SM. These silent BGC may require ecological cues for their stimulation—conditions that are difficult to find or to reproduce in a laboratory. Out of the 24 BGC predicted in the model strain *M. xanthus* DK1622 using the antiSMASH software, only nine of them have been described so far, with seven belonging to the NRPS/PKS family (DKxanthenes, myxoprincomides, myxalamids, myxochelins, myxovirescins, myxochromides, and alkypyrones) (5, 6, 15, 16). Among them, myxalamids (PKS/NRPS), myxochelins (PKS/NRPS), DKxanthenes (PKS/NRPS), and myxochromides (PKS/NRPS) are present in mainly all genomes of *M. xanthus* strains and are detected in the metabolic profile of *M. xanthus* DK1622 (17). For a few of these SM, a physiological function has been associated or identified: the yellow pigments, DKxanthenes, are involved in fruiting body formation and sporulation; myxochelins are siderophores; and myxovirescins have antibiotic properties notably involved in predation (5, 7, 18–21). For most SM, although their physiological role is poorly understood, bioactivities of medical importance (e.g., antibacterial, antitumoral, antifungal, antiproliferative properties) have been detected such as for myxalamids, myxochelins, myxovirescins, and cittilins (3, 7–9, 11, 19, 20, 22–24).

Compared to the well-studied *Streptomyces* and *Bacilli* microorganisms, approaches to activate the production of myxobacterial SM, whose expression of the corresponding BGC is otherwise silent (i.e., cryptic BGC), are still far behind but are in expansion mostly in the model bacteria *M. xanthus* DK1622 or the closely related *M. xanthus* DZ2 (6, 25, 26). Myxobacteria are known to require complex cultivation media; hence, the one strain-many compounds (OSMAC) approach, which consists of growing the same strain in different conditions combined with recent, new analytical and supercritical fluid extraction (SFE), led to the detection, purification, and characterization of myxobacterial SM (15, 26, 27). Recently, in addition to genetic tools used as routine in *M. xanthus* DK1622 or DZ2, constitutive strong promoters have been shown to efficiently enhance the expression of BGC encoding DKxanthenes and myxochromides in *M. xanthus* DK1622, and greatly expand the synthetic biology toolkit available for the *M. xanthus* model strains (28). Homologous or heterologous expression of BGC, thanks to a constitutive promoter, could be effective to produce otherwise cryptic SM in sufficient amount for biophysical and biological activity characterization. However, other limitations might arise such as the availability of required common primary metabolites for the biosynthesis of the cognate SM, especially those of the NRPS/PKS family (29).

Here, we have enriched the toolbox for SM production with (i) a new growth protocol called 2PRIM (two-step Protocol for Resource Integration and Maximization) and (ii) an *M. xanthus* DZ2 chassis strain called BOOST (Biomolecules Overproduction and Optimal Screening Therapeutics) in which the production of the four main NRPS/PKS SM has been abolished. In this study, we show that by combining growth optimization and the chassis strain, we have succeeded in improving the production of the NRPS/PKS myxoprincomide, when expressed under both its natural and a constitutive strong promoter. Interestingly, the strong production level allowed us to annotate new myxoprincomide congeners. Also, our new myxobacterial chassis strain (BOOST) is significantly attenuated for antibacterial and anticancer activities, which is a prerequisite to be exploited for drug screening. Thus, the developed 2PRIM-BOOST approach is of interest for performing biological activity screening and biophysical characterization of otherwise SM from cryptic myxobacterial BGC.

## RESULTS

### The 2PRIM culture protocol allows *M. xanthus* to accumulate molecules involved in biosynthesis of lipids required for secondary metabolite biosynthesis

**Fig 1:**
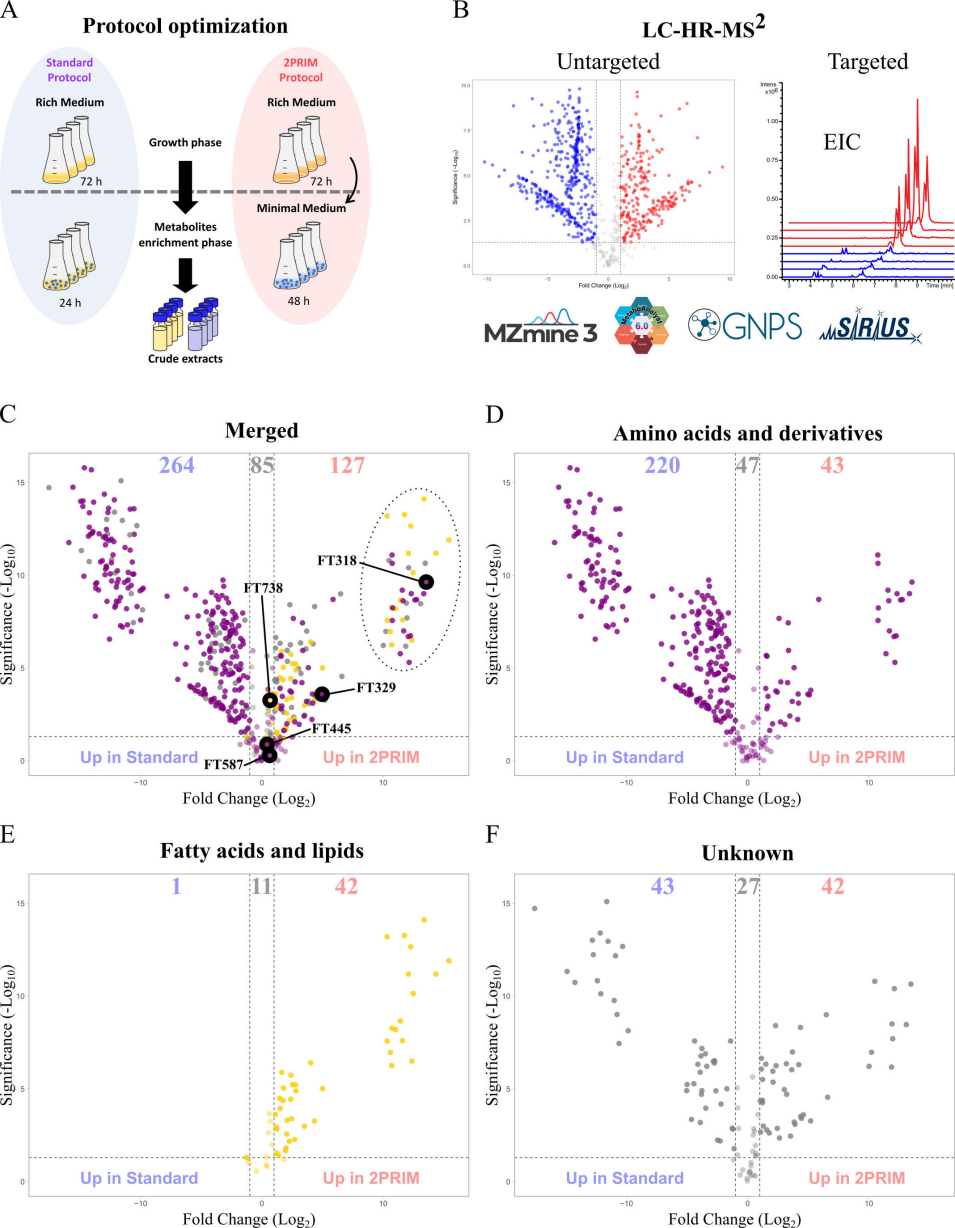
Comparison of the WT metabolomes using the standard and the 2PRIM protocols. (**A**) Description of the standard and the 2PRIM protocols. The protocols are divided into two steps: first, a growth step to produce biomass, and the second, to enrich the culture with metabolites with the addition of 2% of Amberlite XAD16 resin. For the standard protocol (blue oval), the CTT rich medium is used for the two steps. In the 2PRIM protocol (red oval), the biomass production step is performed in CYE rich medium, and cells were transferred in CF minimal medium for the enrichment step. (**B**) Prepared crude extracts of *M. xanthus* DZ2 strain were analyzed by Liquid Chromatography High-Resolution Tandem Mass Spectrometry (LC-HR-MS^2^), followed by untargeted and targeted analysis using MZmine, MetaboAnalyst, GNPS, and SIRIUS. (**C–F**) Volcano plot of data matrix 476FT showing the fold change difference of a given metabolite from the 2PRIM and the standard protocols (2PRIM/standard ratio). (**C**) Merged volcano of D, E, and F. Features with a positive log_2_ fold change value (UP in 2PRIME) or with a negative log_2_ fold change value (UP in standard protocol) above the thresholds (*P* value below 0.05 and fold change above 2) are represented. Some features are highlighted; FT318 corresponds to S-acetyl-pantetheine, FT329 to myxoprincomide c-506, FT445 to cittilin A, FT587 to DKxanthene 534, and FT738 to myxalamid A. The dotted circle surrounds the 41 MS features that are the most produced in 2PRIM relative to the standard protocol. Total volcano plot is decomposed to highlight the amino acids and derivates (purple dots) (**D**), the fatty acids and lipids (yellow dots) (**E**), and unknown metabolites (gray dots) (**F**). Metabolites from the blank medium of each protocol were subtracted. Extracts from each protocol (*n* = 4) were normalized at 5 mg/mL in MeOH.

We reasoned that a culture protocol that better reflects the environmental conditions faced by *M. xanthus* might improve SM production. We then designed a nutritional starvation protocol called 2PRIM (Fig. 1A in red). The 2PRIM consists of two steps, the first one being a growth step in rich medium (casitone-yeast extract, CYE) to accumulate biomass, whereas the second one is performed in minimal medium (colony forming, CF) (Fig. 1A). Untargeted and targeted MS-based metabolomic analyses were performed to further evaluate the metabolic differences between extracts resulting from cultivating *M. xanthus* DZ2 using either the standard or the 2PRIM protocol (Fig. 1B). Data processing on MZmine 3 led to the production of a matrix containing 823 features (*syn*. chemical signals, referred as features, FT), each of them characterized by a chromatographic retention time (RT), mass-to-charge ratio (*m/z*), and the area integrated across the chromatographic peak shape. This data matrix encompasses all the MS-detected signals in each of the *M. xanthus* DZ2 extract replicates minus the signals obtained from the XAD16-treated culture media.

**Fig 2:**
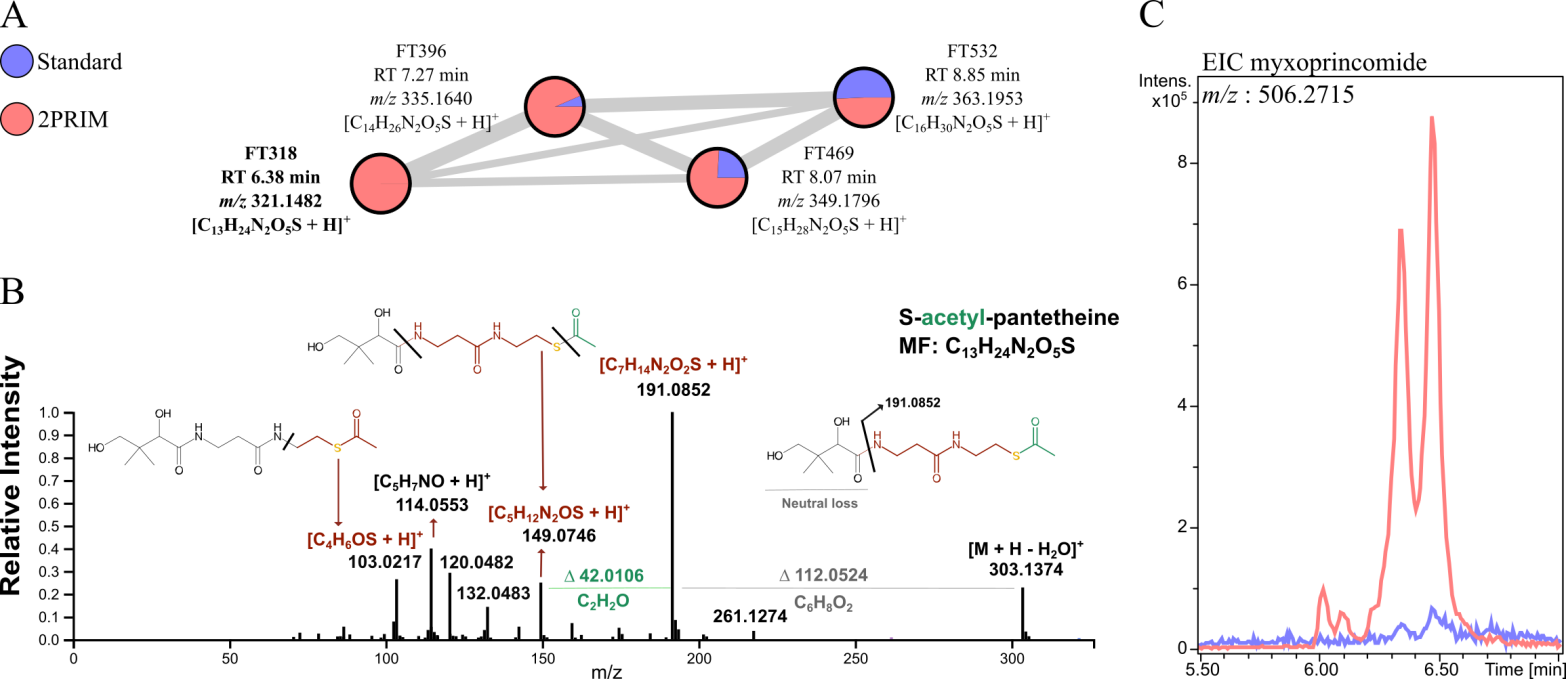
*M. xanthus* accumulates derivates of pantetheine and the myxoprincomide SM in the 2PRIM protocol. (**A**) Analysis of the spectral family 54, identified as thioester derivates of pantetheine, in extracts obtained using the standard and the 2PRIM protocols. Each feature is identified by its RT and *m/*z. The pie chart represents the relative concentration of features in blue for the standard protocol and in red for the 2PRIM protocol. (**B**) MS2 spectrum annotation of FT318 identified as S-acetyl-pantetheine. (**C**) EIC of myxoprincomide c-506 *m/z*: 506.2715 [M + 2H]^2+^. Shown in red is the myxoprincomide EIC from the 2PRIM protocol, and in blue, the one from the standard protocol.

To evaluate the chemical similarities and differences between both cultivation conditions, FT-based molecular network, clustering features in spectral families based on similarities of the fragmentation patterns in their MS^2^ spectra, was generated on the Global NaturalProducts Social Molecular Networking (GNPS) platform. From the 823 data matrix, signals of features that do not cluster in spectral families were removed. Using MetaboAnalyst 5.0, a volcano plot was performed using the resulting annotated data matrix (476 features) in order to highlight and visualize the distribution of the features whose amount was significantly and statistically changed in the 2PRIM condition compared to the standard one (minimum fold change [FC] = 2, *P* < 0.05) (Fig. 1C). Annotation of features was performed using *in silico* tools embedded in SIRIUS, and the proposed structural class of each feature was compared to spectral matches from GNPS libraries using ConCISE. Interestingly, only 85 features were found in the same proportion between extracts originating from both cultivation conditions, whereas 264 and 127 features were more abundant when using the standard and the 2PRIM protocols, respectively (Fig. 1C). The results showed that in the standard protocol, the bacterial metabolome is mainly composed of small peptides and amino acid derivatives (Fig. 1D), whereas bacterial extracts from the 2PRIM protocol contained significantly more fatty acids and lipids (Fig. 1E). Features from the unknown families, which correspond to features without structural superclass consensus prediction given by ConCISE, are homogenously distributed in both protocols (Fig. 1F). Next, we focused our structural identification efforts toward features that were the most produced in the 2PRIM condition relative to the standard protocol (Fig. 1C, upper right circled part). For that purpose, we combined results from *in silico* annotation with manual interpretation of MS^2^ spectra using the generated molecular network as a support (Table S1). From the 41 selected features, a total of 29 molecules were putatively identified (confidence levels 2–3) (Table S1). Most of the molecules were found to be diacyl-glycerol derivatives (10 molecules), three were assigned as triterpenoids, and six of them were annotated as small peptides (Table S1). The feature with the highest fold change among the organic acid structural superclass, FT318 (*m/z* of 321.1482, [M + H]^+^ calculated for C_13_H_25_N_2_O_5_S), retained our attention (Fig. 1C). This feature is directly linked to three other signals on the generated molecular network (Fig. 2A; Table S2). All spectra within this family showed (i) a characteristic fragment at *m/z* = 149.0749 (calculated for [C_5_H_12_N_2_OS + H]^+^ and (ii) a systematic neutral loss calculated for Δ = 112.0524 atomic mass units (amu) and corresponding to C_6_H_8_O_2_ (Fig. 2B; Fig. S1). Such annotation led us to assign this spectral family to pantetheine derivatives. The MS^2^ spectrum of FT318 was also characterized by an intense fragment at *m/z* 191.0852 calculated for [C_7_H_14_N_2_O_2_S + H]^+^ together with the neutral loss of Δ = 42.0106 amu attributed to an acetyl moiety (Fig. 2B). FT318 was, thus, putatively identified as S-acetyl-pantetheine (Fig. 2B). Using the same annotation process, FT396 was putatively identified as S-propionyl-pantetheine, FT469 as S-butyryl-pantetheine, and finally, FT532 as S-isovaleryl-pantetheine (Fig. S1).

Altogether, these results unveil that the 2PRIM protocol permits to obtain a simplified chemical profile and triggers the accumulation of lipid compounds and acyl-pantetheine molecules in *M. xanthus* DZ2.

### Production of NRPS/PKS SM using the 2PRIM protocol

We then looked at the production of the most representative SM of *M. xanthus* strains such as DKxanthenes, myxalamids, myxochelins, myxochromides, myxovirescin, and cittilins in the 2PRIM versus the standard protocol (Table S3; Fig. S2). Notably, FT329 identified as the NRPS/PKS myxoprincomide c-506 (FT329) was significantly more abundant in all extracts from the 2PRIM condition (fold change = 31) (Fig. 1C and 2C). In addition, DKxanthenes 560 (FT628) and 520 (FT582) were also found, in average, more abundant in 2PRIM extracts (Table S1). Myxalamid A (FT738), DKxanthene 534 (FT587), myxovirescin A, and cittilin A (FT445) showed nearly identical abundance in extracts from the two protocols (Fig. 1C). The iron-chelating compounds myxochelins A and B were only detected using the standard protocol, most likely because the medium used in this protocol has low iron content (data not shown). Last, the myxochromides previously detected in *M. xanthus* DK1622 were not detected, whatever the protocol used (17).

To conclude, the 2PRIM protocol strongly favors the production of the NRPS/PKS myxoprincomide.

### Phenotypic and metabolomic characterization of the BOOST chassis strain lacking the four main NRPS/PKS secondary metabolites

**Fig 3:**
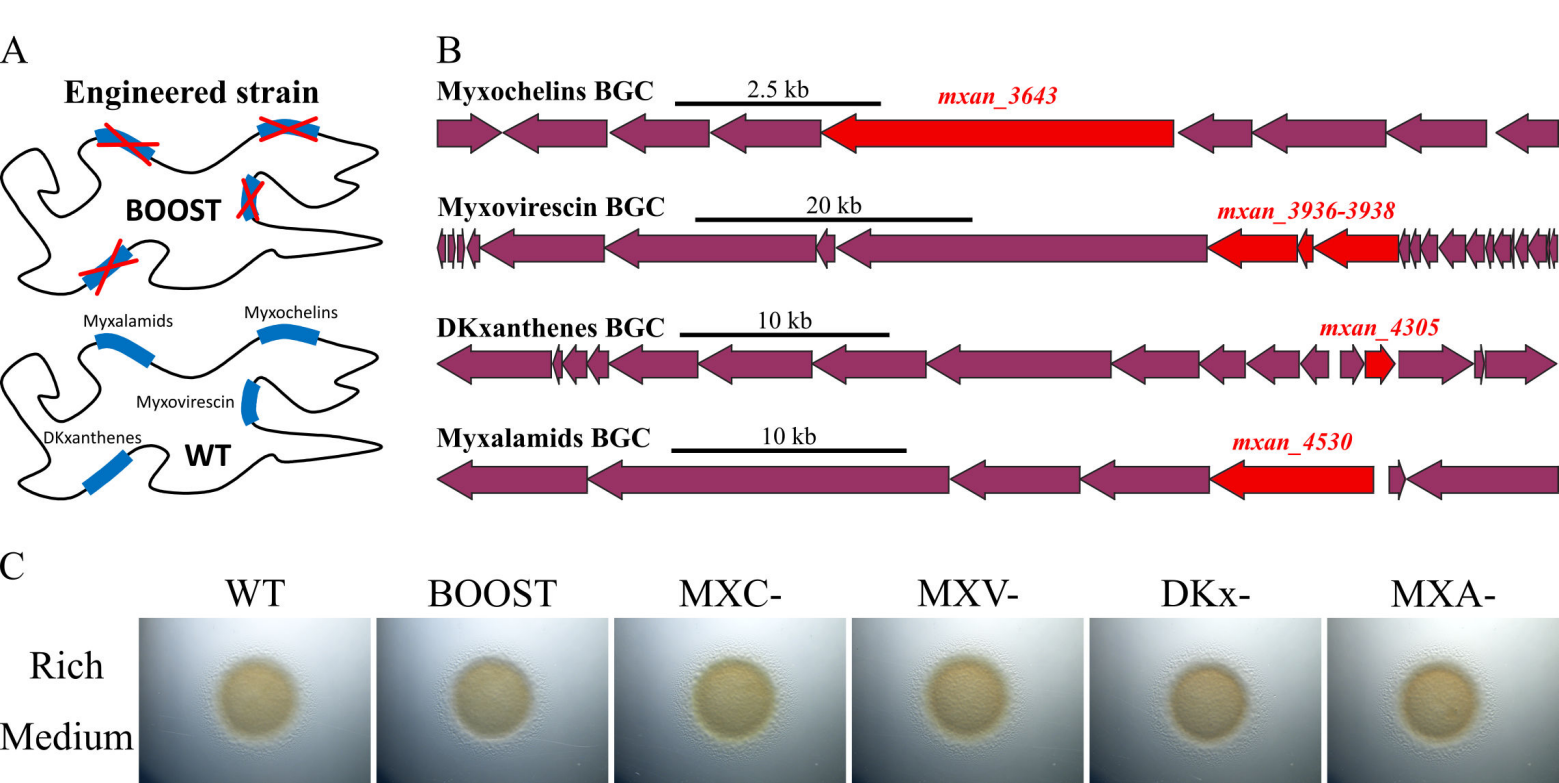
The deletion of the main secondary metabolites does not affect the growth of the BOOST strain. (**A**) Scheme of the genotype of the BOOST chassis strain in which the four BGC encoding the pathways to produce the SM myxalamids, myxochelins, DKxanthenes, and myxovirescin were deleted. (**B**) Genomic structure of BGC encoding myxochelins, myxovirescin, DKxanthenes, and myxalamids. Genes in red were deleted by double recombination. (**C**) Colony morphology and motility of *M. xanthus* DZ2 WT and mutant strains. Pictures of the colonies were taken after 48 h of growth on CYE plates with 1.5% agar. MXC-, myxochelins null strain; DKx-, DKxanthenes null strain; MXV-, myxovirescin null strain; and MXA-, myxalamids null strain.

Next, we engineered an *M. xanthus* DZ2 chassis strain in order to abolish the production of the most abundant and biologically active NRPS/PKS SM (Fig. 3A). Thus, we engineered the BOOST strain by sequentially deleting the targeted genes encoding the NRPS/PKS of the four major secondary metabolites in *M. xanthus* DZ2 (DKxanthenes, myxalamids, myxochelins, and myxovirescin) by a double crossover approach (Fig. 3A and B). The deleted genes were *mxan_3936* to *3938* for the myxovirescin (30), *mxan_4530* for myxalamids (31), *mxan_3643* for myxochelins (32), and *mxan_4305* for DKxanthenes (33) (Fig. 3B).

**Fig 4:**
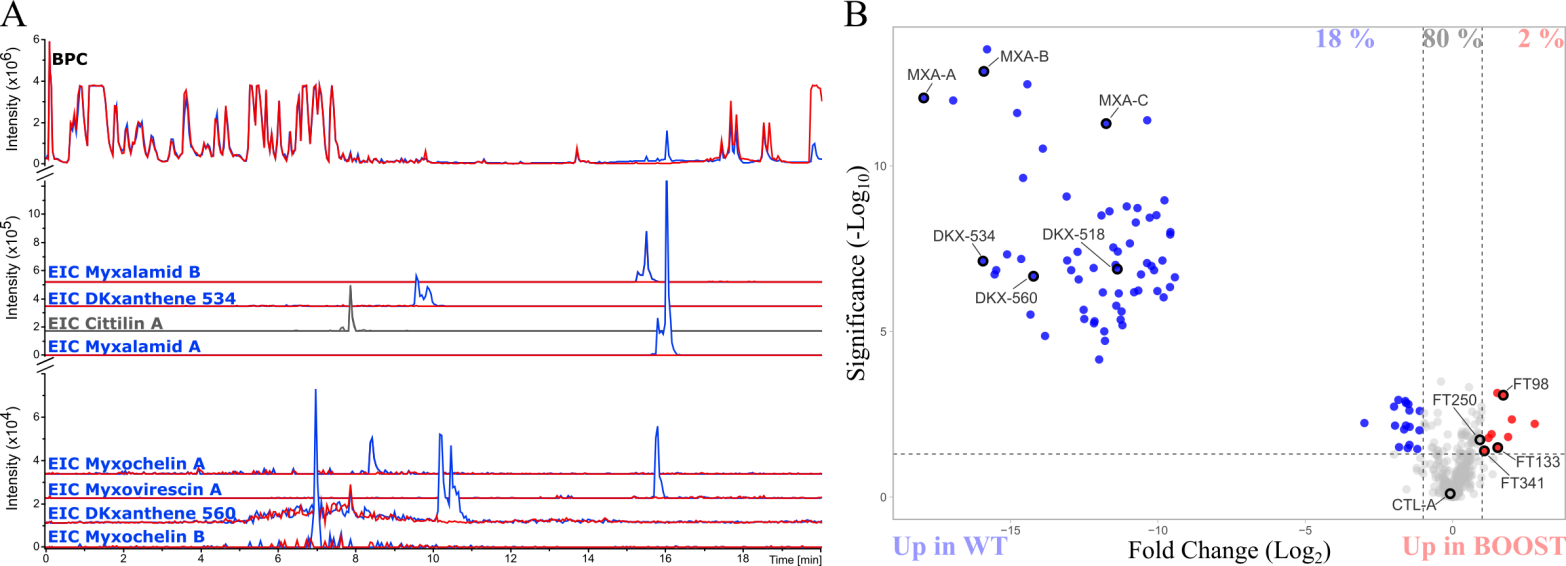
Comparison of the metabolome composition between the BOOST and the WT strains. (**A**) Upper part: comparison of the base peak chromatogram (BPC) of the WT (in blue) and BOOST (in red) strains. Lower part: the EICs of the main SM of *M. xanthus* are highlighted. EIC from the WT strain (in blue) and the BOOST strain (in red). Cittilin A EIC is highlighted in gray for both strains because they have the same intensity. (**B**) Volcano plot showing the fold change difference of an FT between the BOOST and the WT strains following the 2PRIM protocol (BOOST/WT ratio). Features with a positive log_2_ fold change value are more produced in the BOOST strain, and features with a negative log_2_ fold change value are more produced in the WT strain (the threshold was set with a *P* value below 0.05 and a fold change above 2). The FT present in the same proportion for both strains are indicated in gray. DKX, DKxanthene; MXV, myxovirescin; MXA, myxalamid; MXC, myxochelin; CTL, cittilin. Some other FT defined by their cluster index (FTXXX) are also highlighted and described in Table S4. Metabolites from the blank medium of each protocol were subtracted. Extracts from each protocol (*n* = 4) were normalized at 5 mg/mL in MeOH.

We first phenotypically characterized the BOOST strain and showed that the absence of the four secondary metabolites did not influence growth nor colony morphology in CYE rich medium on 1.5% agar (Fig. 3C). Also, the BOOST strain was able to predate *Escherichia coli* as the wild-type (WT) DZ2 strain (Fig. S3). MS-based metabolomic analyses targeting the molecular ions corresponding to the four types of deleted metabolites confirmed that the BOOST strain did not produce DKxanthenes, myxalamids, myxochelins, and myxovirescin (Fig. 4A).

Then, we performed an MS-based metabolomic analysis to compare the metabolic profiles of the BOOST and the WT strains when cultivated following the 2PRIM protocol. The untargeted comparative analysis is presented as an annotated volcano plot (Fig. 4B). Collectively, the features absent in the metabolome of the BOOST strain represent 18% of all detected features in the 2PRIM condition and correspond to analogs or derivatives of the four deleted NRPS/PKS SM. As a control, we checked the amount of an SM that does not result from a NRPS/PKS biosynthetic pathway, the cittilin A. This metabolite was detected in the extract from both strains at the same level of intensity (Fig. 4B). With the exception of the four targeted SM families, extracts from the WT and the BOOST strains showed nearly similar metabolite profiles, with 80% of shared features (Fig. 4B).

Next, we focused our spectral annotation to chemical features (2% of the total features) detected more intensely in extracts from the BOOST strain compared to the WT (Fig. 4B). Among them, three metabolites belonged to the lipids and fatty acids structural superclass with the putative identification of phosphatidylethanolamine derivatives (PE, FT341 in Fig. 4B; Table S4). It is to be noted that FT250, with an *m/z* of 363.1953 ([M + H]^+^ calculated for C_16_H_31_N_2_O_5_S), is close to the fold change threshold of 2 (FC = 1.9) and was putatively identified as S-isovaleryl-pantetheine as described above (Fig. S1). Interestingly, a total of two features, FT98 and FT133, with a respective fold change of 3.3 and 2.9 could not be further annotated beyond the molecular formula and did not match any known compounds in chemical databases (Fig. 4B; Table S4). Altogether, these results allow us to conclude that the chassis BOOST strain shows an accumulation of lipid compounds and a slight accumulation of a thioester derivative of the S-acetyl-pantetheine, the isovaleryl-pantetheine.

In summary, compared to the WT strain, the BOOST strain exhibits a normal growth and a simplified basal metabolic fingerprint without the main known bioactive metabolites. The resulting simplified chemical profile provides a background that will facilitate the detection of newly synthetized and possibly bioactive metabolites.

### The BOOST strain exhibits weaker biological activities than the WT strain

As the purpose of the BOOST chassis strain is to be used to screen biological activity, it was important to verify that it will not impede screening for important biological activities (e.g., antibacterial, antifungal, antiproliferative). We, therefore, ran a series of bioassays.

**Fig 5:**
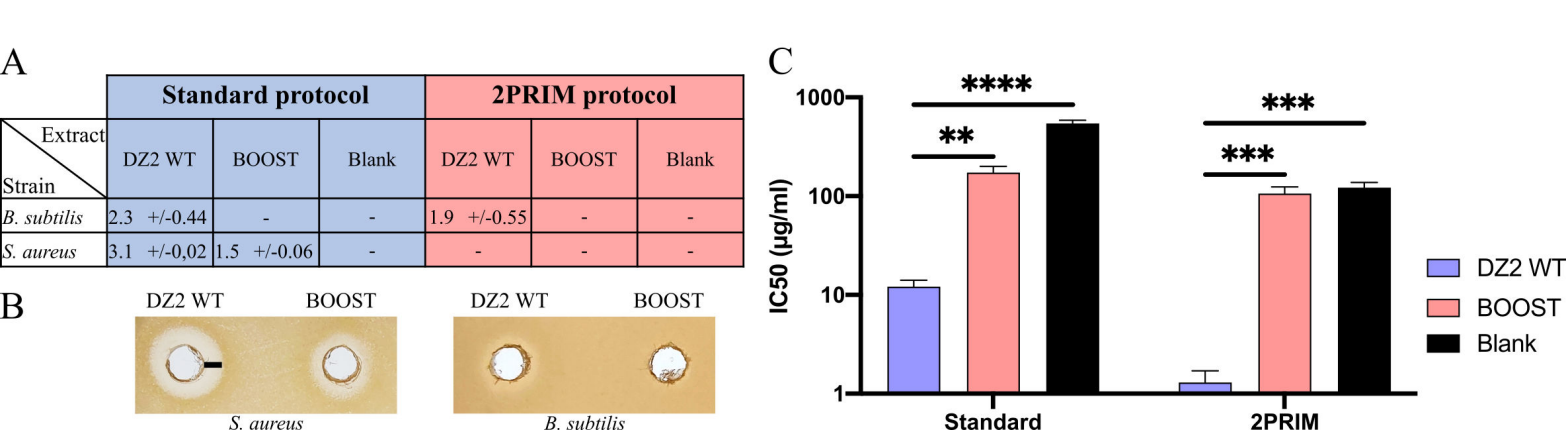
The BOOST strain exhibits a significant loss of biological activities. (**A**) The table sums up results of the inhibition zone of *S. aureus* and *B. subtilis* on agar plates. Size of the inhibition zone is expressed in millimeters and is measured from the border of the well to the limit of the inhibition zone as shown by the black line in panel B. For agar test, 2 mg of dimethyl sulfoxide (DMSO) extracts from the standard protocol and 1 mg of DMSO extracts from the 2PRIM protocol were used. The blank was performed with DMSO only. Standard errors were calculated from two biological replicates. (**B**) The inhibition zones of *M. xanthus* DMSO extracts on LB agar plates against *S. aureus* with the standard protocol extracts and against *B. subtilis* with the 2PRIM extracts. (**C**) The 50% inhibitory concentrations (IC_50_) on the proliferation of human lung cancer epithelial cells (A549 cells) were calculated after exposure to DMSO extracts of *M. xanthus* strains prepared following the standard or 2PRIM protocols. Errors bars show the standard error of three technical replicates, and statistical analysis was performed with a Tukey test (**, *P* < 0.01; ***, *P* < 0.001; ****, *P* < 0.0001; ns, not significant).

Interestingly, no inhibitory activity against *Bacillus subtilis* was observed from the BOOST strain cell extracts, whereas the WT crude extracts from the standard and the 2PRIM protocols led to an inhibition zone of 2.3 and 1.9 mm, respectively (Fig. 5A and B).

Using the standard protocol, crude extracts of the BOOST strain had a twofold decreased inhibitory activity against *Staphylococcus aureus*, compared to the WT DZ2 crude extracts (100 mg/mL) (Fig. 5A and B). No activity of the WT and the BOOST strains were detected against *Candida albicans*, *E. coli*, *Klebsiella pneumoniae*, *Pseudomonas aeruginosa*, *Acinetobacter baumanii*, *Enterobacter cloacae*, and *Enterococcus faecalis* (data not shown).

Then, we looked for the anticancer activity of the crude extracts using A549 human lung cancer epithelial cells as model. After 48 h of exposure, WT strains showed a strong antiproliferative activity with a 50% inhibitory concentration (IC_50_) of 12.1 µg/mL for the standard condition extracts and of 1.3 µg/mL when extracts were prepared with the 2PRIM protocol. In contrast, extracts from the BOOST strain did not show any significant activity (Fig. 5C, results similar to the blank).

These results pinpoint that the BOOST chassis strain exhibits weaker anticancer and antibactericidal activities than the WT strain. Therefore, the BOOST strain could enable a straightforward biological activity and chemical identification of new SM whose biosynthesis has been induced.

### Proof of concept of the 2PRIM-BOOST approach using myxoprincomide

**Fig 6:**
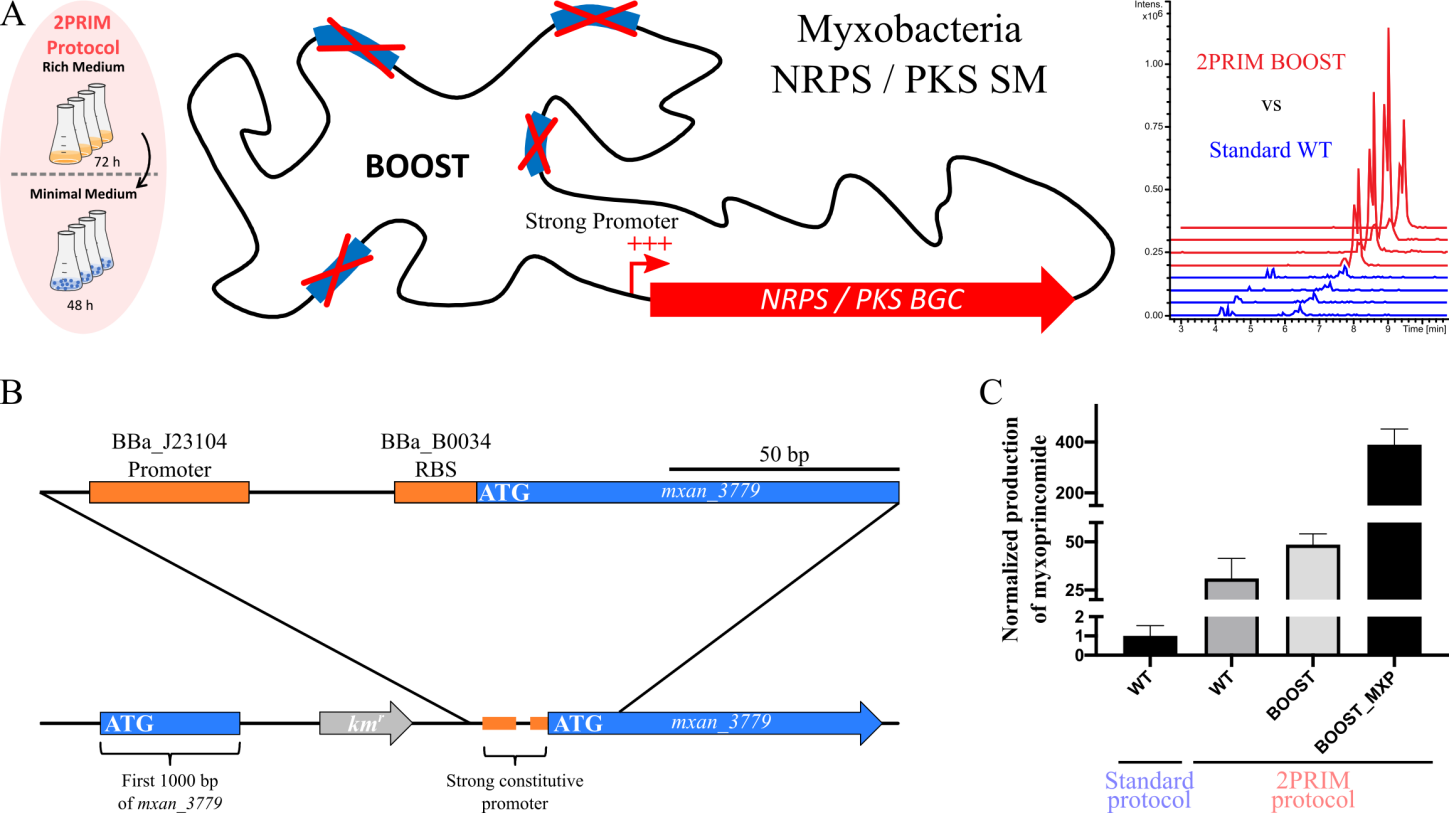
The combination of 2PRIM-BOOST and a constitutive strong promoter highly enhances the production of myxoprincomide. (**A**) Scheme of the 2PRIM-BOOST concept to produce cryptic or weakly produce SM by combining the 2PRIM protocol to the BOOST strain with the insertion of a constitutive strong promoter to induce the expression of the desired BGC and the corresponding SM. (**B**) Scheme of the construction to express myxoprincomide BGC (*mxan_3779*) with the synthetic strong promoter BBA_J23104 and the RBS BBa_B0034 inserted into the locus by a single step of homologous recombination. (**C**) The production of myxoprincomide c-506 is normalized to the production in the WT strain calibrated to 1 following the standard protocol. The myxoprincomide c-506 amount following the 2PRIM protocol was calculated by comparison of the integration of each EIC from WT, BOOST, and BOOST_MXP strain extracts. The proportional correlation of the integration of the EIC of myxoprincomide c-506 was verified by making a standard curve from diluted extracts of the BOOST_MXP strain. Error bars show the standard error of four biological replicates.

As a proof of concept, to test whether the BOOST chassis strain cultivated following the 2PRIM protocol can favor the production of a known weakly produced NRPS/PKS SM, we chose the NRPS/PKS secondary metabolite myxoprincomide (Fig. 6A) (34, 35). We showed above that myxoprincomide production in the WT strain was significantly more abundant in extracts obtained using the 2PRIM condition compared to the standard condition (fold change = 31) (Fig. 1C, 2C, and 6C). This was determined after UHPLC-HR-MS analysis by measuring the area under the curve (AUC) of myxoprincomide c-506 extracted ion chromatograms (EIC *m/z* = 506.2715) in each analyzed extract and reported to a calibration curve (Fig. S4; Table S5).

When combining the BOOST strain and the 2PRIM conditions, we obtained an increased amount of myxoprincomide (fold change = 48) compared to the WT strain grown in the standard condition (Fig. 6C).

Constitutive synthetic promoters and ribosome binding sites have already been shown to be efficient tools to improve SM production (28). We, therefore, have combined these tools to those developed here (BOOST/2PRIM) and applied them to produce myxoprincomide. The synthetic strong promoter J23104, followed by the RBS B0034 (28), was inserted at the locus to overexpress *mxan_3779*, giving the resulting BOOST_MXP strain (Fig. 6B). Overall, the combination of a synthetic strong promoter, the BOOST chassis, and the 2PRIM protocol led to an increased production of myxoprincomide c-506 by a factor of 390 compared to the amount obtained from the WT strain cultivated in the standard condition (Fig. 6C).

Such overproduction was also associated with the generation of a variety (40 features out of 311) of myxoprincomide congeners that represent 12.9% of the detected FT as observed in the generated molecular network (Fig. S5). Some of them were previously reported (36), others are described here for the first time (Table S6; Fig. S6). Such generated structural diversity could serve as a starting point to further evaluate the ecological and/or pharmacological properties of myxoprincomide metabolites.

Collectively, the results obtained with myxoprincomide show that, whether or not myxoprincomide expression is driven by a synthetic promoter, the 2PRIM-BOOST strategy favors the detection of metabolites originally produced at low levels, and paves the way for future investigations aim at enhancing the production of cryptic BGC in *M. xanthus*.

## DISCUSSION

Previous strategies used to produce SM in myxobacteria mainly relied on two main strategies, the OSMAC (5, 15, 37, 38) and the heterologous production in combination with strong constitutive promoters in the model organism *M. xanthus* DK1622 (3, 5, 11, 27, 28, 34, 39–42). These strategies led to the identification of numerous metabolites, yet they merely scratch the surface, with the vast majority of myxobacterial SM still remaining to be discovered. Our work presented here describes two new tools that, in combination with the use of a strong constitutive promoter, will improve the production of silent BGC and will facilitate the screening for biological activities (28). The first tool is a two-step growth protocol for *M. xanthus*, herein called 2PRIM. This protocol was designed to mimic the low-nutrient environment typically encountered by *M. xanthus*. The second tool is the construction of an *M. xanthus* DZ2 chassis strain, herein named the BOOST strain, which lacks the most abundant PKS/NRPS SM.

To design the 2PRIM protocol, we used a minimal medium commonly utilized for *M. xanthus* strains. However, in minimal medium, *M. xanthus* stops dividing and limited amount of biomass is obtained. Thus, we paid attention to include in the 2PRIM protocol a first phase of growth in a rich medium, which is absolutely required to obtain cell biomass. When applying the 2PRIM protocol, we showed in *M. xanthus* (i) an increased amount of S-acetyl-pantetheine and thioester derivates, and (ii) an enrichment of lipids and terpenoids (43–45). Interestingly, these results are in agreement with the fact that transcriptomic data have shown a profound change in *M. xanthus* lipid metabolism when grown in minimal medium (46). Given the fact that the production of PKS/NRPS secondary metabolites is intrinsically connected to lipid metabolism, the 2PRIM protocol might favor the accumulation of building block molecules involved in SM biosynthesis, such as in the case of myxoprincomide biosynthesis that involves malonyl-CoA.

Heterologous production of myxobacterial SM with *M. xanthus* as cell factory is extensively used to express exogenous BGC from strains exhibiting a slow growth rate or genetically unattractable (5, 6, 6, 34, 47, 48). SM such as epothilone, corallopyronins, argyrins, cystobactamids, haliangicin, myxarylins, pyxidicyclines, sorangibactins, myxopyronins, bengamide, dawenol, pretubulysin, PUFAs, vioprolides, disorazole, and myxofacyclines from soil and marine myxobacteria have been heterologously produced with success in *M. xanthus* DK1622 or DZ2 (39, 49–52). All genetic tools have been developed in *M. xanthus* DK1622, and this model bacterium possesses all the common types of biosynthesis machineries necessary for the production of the different classes of SM. We chose to optimize *M. xanthus* DZ2, whose genotype is very close to *M. xanthus* DK1622, as chassis strain because *M. xanthus* DZ2 possesses all qualities described for *M. xanthus* DK1622 and exhibits a much slower cell lysis than DK1622 after 30 h of growing . This is a major advantage for the production of SM that necessitates long culture times (53). As in *M. xanthus* DK1622, *M. xanthus* DZ2 contains DKxanthenes, myxalamids, myxochelins, and myxovirescin (PKS/NRPS), which all belong to the PKS/NRPS family and are the main molecules detected in the metabolomic profile of *M. xanthus* DZ2 in the standard protocol. These four molecules, purified from different myxobacteria, have been shown to exhibit biological activities. DKxanthenes and myxalamids exhibit antifungal activity (8, 11, 27, 54), and myxovirescin has an antibacterial activity by interfering with cell wall synthesis and inhibiting type 2 signal peptidase LspA (8, 11, 20, 24, 55, 56). Myxochelin exhibits antibacterial, antiviral, antitumor, and antiproliferative activities (8, 11, 19, 23, 32, 40). Removing the four main NRPS/PKS secondary metabolites from *M. xanthus* DZ2 allowed us to obtain a mutant strain, referred as BOOST strain, which exhibits valuable characteristics for screening the biological activities of newly produced secondary metabolites. In fact, the BOOST strain (i) presents a simplified metabolite profile, (ii) has a loss of biological activities, and (iii) accumulates a pool of specific precursors required for NRPS/PKS synthesis.

Furthermore, we validated the benefit of combining both the engineered BOOST strain and the 2PRIM protocol, together with the use of a constitutive strong promoter to trigger the expression of a biosynthetic gene cluster. Hence, as a proof of concept, we used the myxoprincomide, an NRPS/PKS SM well characterized but weakly produced in *M. xanthus* DZ2 in the standard protocol (34, 35). Our 2PRIM-BOOST approach combined with the use of the J23104 synthetic strong promoter allows us to obtain an increase of 400 times the amount of myxoprincomide, showing the efficiency of combining strategies. We showed that using the 2PRIM protocol only, myxoprincomide production is approximately 30 times higher. In the study of Cortina et al., they obtained a 30-fold increase in myxoprincomide production by inserting a T7 promoter upstream of the BGC in the *M. xanthus* A2 strain (34), compared to the WT myxoprincomide production in *M. xanthus* DK1622. Interestingly, the higher production of myxoprincomide reached in this study led also to the detection of new congeneric molecules that may hold different biological properties. Such outcomes could serve as a starting point for better understanding of the biological functions of myxoprincomides, similar to investigations into structure–activity relationships.

Using an organism sharing the same metabolic pathway is key to get active heterologous-produced SM from the same phylum. Fast-growing, genetically manipulable, precursor-abundant, simplified metabolite profile and low biological activities of medical interest are all the advantages of the BOOST strain for heterologous expression of BGC from other myxobacteria (slow growing, genetically non-manipulable and uncultivable). From our results, we conclude that the use of the BOOST chassis strain could be an added value to produce yet uncharacterized NRPS/PKS metabolites of myxobacteria (10). Undoubtedly, boosting the production of novel metabolites in bacterial extracts will facilitate their downstream isolation, necessary to confirm their structural identity and biological properties.

Removing BGC from various *Streptomyces* strains has been shown to be a strategy of choice to successfully enhance the production of actinobacterial SM (57–65). SM-free *Streptomyces albus* chassis strains have been constructed in this goal (60), but it appears that the various chassis strains exhibit preferences for producing SM and that BGC can lead to different molecule structures depending on the chassis strains used (57). Additionally, there is a need to construct fast-growing *Streptomyces* chassis strains. Thus, construction of new *Streptomyces* chassis strains is still the subject of particular attention in order to increase the chances of characterizing new SM (61, 66–68). The BOOST strain is the first genome-reduced chassis strain engineered in *M. xanthus* that we showed to be efficient to produce an NRPS/PKS family compound. Whether other types of SM can also be overproduced in the BOOST strain remains to be tested.

Reviews by Bader et al. in 2020 and more recently by Wang et al. in 2024 (5, 6) listed the main strategies used to date for SM identification (bioactivity-guided, cultivation-based, metabolome-based, and genome-based approaches) and highlighted the need to combine several methods to have access to new SM. Here, we further strengthen this notion and propose that the 2PRIM-BOOST approach combined with other transcriptional activation-based methods would be instrumental to increase the chances to discover new microbial metabolites of medical and industrial interests.

## MATERIALS AND METHODS

### Bacterial strains and growth conditions

Strains used in this study are listed in Table S7. *M. xanthus* strains were grown at 32°C on agar plates or in liquid medium on a rotary shaker at 160 rpm for common use and at 30°C and 180 rpm for mass spectrometry experiments. CYE (69), CF medium (70), and the complex (CTT) medium (71) were used as media. Plates contained 1.5% of agar. Kanamycin at 100 µg/mL and galactose at 2.5% (wt/vol) were added to media for selection when specified. *E. coli* strains were grown at 37°C on lysogeny broth (LB) agar plates or in LB liquid medium on a rotary shaker at 160 rpm. When necessary, kanamycin was added at 30 µg/mL.

For strains used to perform the agar well-cut diffusion method, *E. coli*, *B. subtilis*, *P. aeruginosa*, *K. pneumoniae*, *S. aureus*, *A. baumanii*, and *C. albicans* were grown aerobically at 37°C, and *E. faecalis* and *E. cloacae* were grown in micro-aerobic condition at 37°C using GasPak unit.

### Strains and plasmids construction

The BOOST strain was constructed by sequentially deleting the targeted genes encoding the NRPS/PKS of each BGC responsible for the production of the four major secondary metabolites in *M. xanthus* DZ2 by a double crossover approach. The deleted genes were *mxan_3936* to *3938* for the myxovirescin (33), *mxan_4530* for myxalamids (34), *mxan_3643* for myxochelins (35), and *mxan_4305* for DKxanthenes (36). Plasmids used for the construction of the BOOST strain are listed in Table S8. To construct the *M. xanthus* simple mutant of each metabolite by in-frame deletion, 800 bp upstream and downstream of the gene(s) targeted for deletion were amplified by PCR and ligated into the pBJ114 following the hot fusion protocol (50). Primers used are listed in Table S9. The resulting plasmids (pBJ114-∆*mxan3936-3938*, pBJ114-∆*mxan4530*, pBJ114-∆*mxan3643*, and pBJ114-∆*mxan4305*) were first sequenced and then introduced into *M. xanthus* DZ2 strain by electroporation, and the first homologous recombination was selected on agar plates containing 100 µg/mL of kanamycin. The second homologous recombination allowing gene excision and loss of *galK* was selected on agar plates containing 2.5% of galactose (72). To construct the BOOST strain, we first deleted the *mxan4305* gene (strain DKx-), then *mxan4530* (strain ∆2), ∆*mxan3643* (strain ∆3), and finished with the deletion of *mxan3936-3938*. The deletion of each of these genes was confirmed by PCR with the appropriate primers. To check the deletion of the *mxan_4530* gene, we used the 114_hindIII_up4530_for and 114_XbaI_down4530_rev primers used for the plasmid construction, resulting in a 1.6-kb fragment and a pair of primers inside the deleted gene (MXAN_4530_for/MXAN_4530_rev). The same strategy was used to verify each of the introduced mutations (primers are listed in Table S9).

To construct the overproducing BOOST_MXP strain with the J23104 strong synthetic promoter and an optimized RBS BBa_B0034, an initial plasmid pBJ114_SP was constructed. Briefly, two primers (Int_SP_pbj114_For and Int_SP_pbj114_Rev) were designed to include the strong promoter sequence as well as the RBS. Then, these two primers were ligated into the pBJ114 following the hot fusion protocol (50). Then, the first 1,000 bp of *mxan_3779* were amplified by PCR and ligated into the pBJ114_SP previously digested by the appropriate restriction enzymes using a T4 DNA ligase. The sequence of the resulting plasmid pBJ114_SP_*mxan_3779* was first confirmed by sequencing prior to being integrated at the locus site in the BOOST strain by site-specific integration. The resulting BOOST_MXP strain was selected on kanamycin agar plates, and insertion of the plasmid in the genome was confirmed by PCR with the pair of primers 78_for and MXAN_3779_1000pbATG_rev, resulting in a 1.2-kb fragment when the plasmid was integrated.

### *M. xanthus* mobility assay on agar plates

Motility was tested using overnight *M. xanthus* cultures grown at 32°C in CYE medium that were concentrated to an optical density at 600 nm (OD_600nm_) of 5 in CYE. Cells (10 µL) were spotted onto CYE 1.5% agar plates. Agar plates were incubated for 48 h at 32°C.

For the predation assays, from an overnight culture of *M. xanthus* grown at 32°C in CYE medium, cells were washed three times in CF medium and were resuspended at a final OD_600nm_ of 2 in CF medium. For *E. coli* cells, from an overnight culture at 37°C in LB, cells were diluted at a final OD_600nm_ of 2 in LB. Then, 10 µL of *M. xanthus* and *E. coli* was spotted at 8 mm from each other on CF agar plate. Agar plates were incubated for 72 h at 32°C. All pictures of the colonies were taken using the Stereo Microscope Nikon SMZ745T binocular loupe equipped with a Nikon Camera.

### Culture of *M. xanthus* for mass spectrometry extracts

For the standard protocol, cultures were started (T0) with 100 mL of normalized culture at an OD_600nm_ of 0.1 in CTT rich medium in a 500-mL flask. Cultures were grown for 72 h at 30°C under agitation at 180 rpm, and 2% wt/vol Amberlite XAD16 (Sigma-Aldrich) was added to the culture at T72. After an extra 24 h of culture (T96), cells and XAD16 were pelleted for 30 min at 7,000 rpm at room temperature and were washed three times in H_2_O with the same parameters. For analytical purpose (see below), samples of culture medium without bacteria were prepared to be used as blanks. The blank CTT was prepared following the same protocol, except that no cells are present in the medium. For the 2PRIM protocol, the cultures were performed in CYE rich medium instead of in CTT. After 72 h of growth in rich medium, under agitation (180 rpm) at 30°C, cells were pelleted for 30 min at 6,000 rpm and were washed once in CF minimal medium. Then, the cells were resuspended in 100-mL CF minimal medium in 500-mL flasks, and 2% wt/vol Amberlite XAD16 was added in the medium. After 48 h of culture (T120), cells and XAD16 were pelleted and followed the same process as described above. The analytical blank of the minimal medium was prepared as follows: after the first 72 h, we assumed that during the cell wash, approximately 2 × 2 mL of the supernatant stayed in the 50-mL Falcon, so 4 mL of CYE rich medium was mixed with 100 mL of CF minimal medium to simulate the cell wash step. A total of 4 mL of this mixture was again added in 100 mL of CF medium in which the Amberlite XAD16 was added. The last step was the same as for the cell cultures. For all strains, four biological replicates were prepared.

### Preparation of crude extracts of *M. xanthus*

Metabolites from both collected cells and XAD16 pellet were extracted with 2 mL of dichloromethane LC grade (preparative HPLC stabilized with ethanol, CarloErba), to which 10 mL of methanol (MeOH anhydrous grade, CarloErba) was added after 15 min of incubation at room temperature. The solution was vortexed, sonicated for 1.30 min at 40 KHz (TUC-70, Jenken) and vortexed , then spun before collecting the supernatant. The pellet was then extracted again with 10 mL of ethyl-acetate (preparative HPLC-Reag.Ph.Eur, CarloErba) following the same vortex and sonication cycle. All collected supernatants were pooled (~22 mL) to produce one bacterial extract, filtered on glass microfiber filters (0.7–2.7 µm MF200 47 mm, Fisher Scientific) to remove cellular particulates, and then concentrated to dryness (MiVAc Quattro, Genevac). Dried extracts were resuspended in LC-MS grade MeOH at a final concentration of 5 mg/mL, centrifuged at 14,000 rpm (5 min at −4°C, SIGMA 1-14K), and then filtered on PTFE luer-lock filter (0.22 µm, cat#26142, Restek) prior to MS analyses. Extracts from myxoprincomide overproducing strain were prepared at 0.5 mg/mL. For downstream LC-MS analyses, quality control samples (*syn*. Pool) were prepared by mixing an equal volume of extracts analyzed in the same acquisition sequence.

### UHPLC-UV HR-MS acquisition

LC-MS metabolomic analyses were performed on a Thermo Scientific Dionex Ultimate 3000 UHPLC system equipped with a diode array detector and connected to an electrospray-ionization-quadrupole time-of flight (ESI-Q-TOF) IMPACT II mass spectrometer (software Bruker OtofControl version 5.3). Chromatographic separation was achieved using a Luna Omega Polar C18 UHPLC 1.6 µm 100 Å column (150 × 2.1 mm, Phenomenex, USA), maintained at 42°C, with an elution gradient composed of (A) water and (B) acetonitrile both with 0.1% formic acid, under the following conditions: from 5% (B) during 7 min to 44% (B) at 7 min, to 50% (B) at 10 min, then to 95% (B) at 15.5 min and during 4.5 min (flow rate of 0.45 mL min^−1^, injection volume 2 µL). Mass spectrometry detection parameters in ESI positive mode were set as follows: nebulizer gas N_2_ at 3.5 bars, dry gas at 12 L min^−1^, capillary temperature at 200°C, and voltage at 4,500 V. MS/MS acquisition mode was set with a scan rate of 4 Hz (full scan from 50 to 1,200 *m/z*) and a mixed collision energy of 20–40 eV (50% time at each collision energy, stepping mode). A sodium formate/acetate solution forming clusters on the studied mass range was used as calibrant and was automatically injected before each sample for internal mass calibration, ensuring a precision of *m/z* lower than 2 ppm on the mass range. Extracts were randomly injected to integrate any memory effect on the column and time-dependent MS drift. Pool samples injected every six samples from the beginning to the end of the series were used for further ion filtering.

### UHPLC-MS data processing

Following their calibration, the acquired MS data were converted to the open format *.mzXML using MSConvert (Proteowizard) (73) and were further processed on MZmine 3.3 (74) for feature detection, as follows : (i) mass detection (centroid, MS1 noise level 500, and MS^2^ noise level 500), (ii) ADAP chromatogram builder (four scans, group intensity threshold 500, minimum highest intensity 500, *m/z* tolerance 10 ppm), (iii) chromatogram resolving (baseline resolver: minimum peak height 20,000, peak duration range 0.0–1.0 min, baseline level 5,000, RT range for MS^2^ scan pairing 0.4 min, number of data point 2), (iv) isotopic grouper (13C isotope filter: *m/z* tolerance 10 ppm, RT tolerance 0.1 min, representative isotope most intense), (v) join aligner was used to align features retention times (*m/z* tolerance 10 ppm, weight for *m/z* 75%, weight for RT 25%, RT tolerance 0.1 min), (vi) feature list rows filter (RT 0.5 to 23 min; keep only peaks with MS^2^ scans and features reproducibly detected in all replicates), and (vii) feature list blank subtraction (minimum of detection in blank 1 and fold change increase of 300%). Features detected only in MeOH as well as features corresponding to culture media and to XAD16 contaminations were subtracted.

MS data corresponding to the comparison of cultivation protocols led to generation of a data matrix with 823 FT. The data matrix produced from the comparison of WT and BOOST strain metabolic profiles contained 435 FT. Finally, all MS data processing pertaining to the analysis of myxoprincomide-modified strains followed the same steps except for (iii): chromatogram resolving (baseline resolver: minimum peak height 5,000, peak duration range 0.0–1.0 min, baseline level 1,000, RT range for MS^2^ scan pairing 0.4 min, number of data point 2). All feature lists and their corresponding list of MS^2^ spectra (mgf file format) were exported for molecular networking, *in silico-*based annotations and statistical analyses.

### Molecular networking and analysis of chemical diversity

Further analysis of spectral similarities and overall MS detectable chemical diversities were performed through Feature Based Molecular Networking on the GNPS platform (https://gnps.ucsd.edu) (75). The precursor ion mass tolerance was set to 0.02 Da and the MS/MS fragment ion tolerance to 0.02 Da. A molecular network was created where edges were filtered to have a cosine score above 0.7 and more than six matched peaks between MS^2^ spectra. Furthermore, edges between two nodes were only kept in the network if each of the nodes appeared in each other’s respective top 10 most similar nodes. Finally, the maximal size of a molecular family was set at 100, and the lowest scoring edges were removed from molecular families until the molecular family size was below this threshold. The resulting network was visualized and interpreted using Cytoscape 3.9.1 (76). GNPS jobs of the different analyses are available following the given links: for comparison of cultivation conditions (standard vs 2PRIM, 823 FT): https://gnps.ucsd.edu/ProteoSAFe/status.jsp?task=cd2673eb70ea47aa9561d6902ec36aee; for comparison of WT vs BOOST strain (435 FT): https://gnps.ucsd.edu/ProteoSAFe/status.jsp?task=9a7b4f773d734045ae880cb5ebdd34e6; and for metabolome of BOOST_MXP strain (311 FT): https://gnps.ucsd.edu/ProteoSAFe/status.jsp?task=c5126a425d894bec8bf94e7219a8c1f1. Table metadata are available in Table S10.

### MS data annotation and dereplication

The MZmine exported mgf file containing MS1 and MS^2^ spectral information for each detected features was processed using *in silico* software embedded in SIRIUS 5.6.3 (77). Higher confidence assignment of molecular formulas for features within each spectral families and structural predictions was performed using SIRIUS-linked and in-house databases built with simplified molecular-input line-entry system (SMILES). *Myxococcus*-related molecular database was developed with data exported from Lotus Natural Product database (78). CANOPUS compound summary file containing systematic annotation of compound classes of selected structural predictions was then exported. ConCISE (Consensus Classifications of *in silico* Elucidations) was run to leverage the *in silico* CANOPUS annotations with matches obtained through GNPS spectral libraries, thereby increasing the confidence in spectral annotations. Chemical distribution and classification of clustered features were performed using outcomes from ConCISE and NPclassifier, using a natural product pathway probability of >0.7. MS spectra from selected molecules of unknown identity but with a proposition of molecular formula were also subjected to MetFrag (79). Targeted MS spectra were manually inspected and further annotated. All previously reported *M. xanthus* secondary metabolites were dereplicated by comparing their MS data with those reported in the literature (34, 54, 80–83).

### Relative proportion of metabolites in bacterial extracts

#### Comparison of the 2PRIM and standard protocols

The exported MZmine data matrix (823 FT) annotated through SIRIUS and using ConCISE was further processed to remove all single nodes (FT, not considered by ConCISE and not clustering in spectral families). The newly generated FT list (476 FT) was submitted to MetaboAnalyst 5.0 (84) web application. MS data were log transformed and autoscaled (mean centered and divided by the standard deviation of each chemical feature) prior to statistical analysis. A volcano plot representing significant fold changes between conditions was generated using the following selection criteria: minimum FC of 2 and statistical significance using a *t*-test (*P* value < 0.05). Data from the volcano plot were exported to be visualized in VolcaNoseR (85).

#### Comparison of myxoprincomide overproducing strains

To determine the relative concentration of myxoprincomide c-506 in the different extracts, a calibration curve was plotted using the AUC of their EICs measured from MS1 acquisition within the same sample matrix analyzed at five different concentrations (from 0.05 up to 5 mg/mL) (Fig. S4). The generated calibration curves were used to calculate the relative concentration in the BOOST, BOOST_MXP, and WT extracts prepared in the 2PRIM (Table S5).

### Measurement of antibacterial activities

The antibacterial properties were measured on LB agar plates following the agar well-cut diffusion method. For bacterial strains, from an overnight culture grown at 37°C in Mueller Hinton (MH) medium, cells were diluted in MH to start a log phase growth and collect cells at a final OD_600nm_ of 1. For *C. albicans*, colonies were harvested from the agar plate and resuspended in MH medium to an approximate OD_600nm_ of 1. Then, cells were diluted in 10 mL of MH at a final concentration between 10^6^ and 10^7^ cells. Plates were flooded with this 10-mL cell suspension during 10 min to let the cells settle down; the suspension was removed, and the plates were left open to dry during 10 min. Subsequently, wells were cut using a tip of 6 mm of diameter, and 20 µL of extract at 50mg/mL for 2PRIM and 100mg/mL for standard was spotted into each well. Plates were incubated for 24 h at 37°C and were photographed with an iPad on a light table.

### Measurement of anticancer activities

The antiproliferative effects of extracts were determined as previously described (doi: 10.3390/biom12060770). Human cancer cells used in this experiment were lung cancer epithelial cells A549 (obtained from ATCC). The cells were routinely maintained on 75-cm^2^ flasks in DMEM supplemented with 10% fetal bovine serum (FBS), 1% L-glutamine, and 1% antibiotics (all from Invitrogen) at 37°C in 5% CO_2_ incubator. For antiproliferative assay, the cells were detached from the flasks using trypsin-EDTA solution (from Thermo Fisher), counted using Malassez chamber and diluted in culture medium before seeding into 96-well cell culture plates (Greiner Bio-One, Paris, France) at approximately 3,000 cells/well. The cells were left for 24 h before treatment with increasing concentration of extracts diluted in culture medium (1:2 serial dilution, from 500 to 0.49 µg/mL for 2PRIM extracts and 1000 to 0.98 µg/mL for standard extracts). After 72 h, the medium was aspirated, and the number of viable cells was determined using a resazurin-based assay. The fluorescence intensity was measured using a microplate reader (Biotek, Synergy Mx) (excitation wavelength of 530 nm/emission wavelength of 590 nm). The fluorescence values were normalized by the controls (untreated cells) and were expressed as percent of proliferation. The IC_50_ values of extracts (i.e., the concentrations causing 50% inhibition of cell proliferation) were determined using the GraphPad Prism 7 software.

## Data Availability

Raw MS data (*.mzXML) are freely available from the UCSD Center for Computational Mass Spectrometry database with the MassIVE identifier MSV000095859.

## SUPPLEMENTAL MATERIAL

Supplemental figures and tables spectrum.01740-24-s0001.pdf Fig. S1 to S6; Tables S1 to S10.

ASM does not own the copyrights to Supplemental Material that may be linked to, or accessed through, an article. The authors have granted ASM a non-exclusive, world-wide license to publish the Supplemental Material files. Please contact the corresponding author directly for reuse.
