# Supplementary material for: Combining culture optimization and synthetic biology to improve production and detection of secondary metabolites in Myxococcus xanthus: application to myxoprincomide
Source: Microbiol Spectr. 2024 Oct 21;12(12):e01740-24. doi: 10.1128/spectrum.01740-24 (PMC11619377; doi:10.1128/spectrum.01740-24)
Supplement: Supplemental figures and tables — Fig. S1 to S6; Tables S1 to S10. [file spectrum.01740-24-s0001.pdf]

Supplementary information:

Combining culture optimization and synthetic biology to improve production and detection of secondary metabolites in *Myxococcus xanthus*: application to myxoprincomide

Mathieu Sourice[a], Charlotte Simmler[b], Marc Maresca[c], Béatrice Py[a] and Corinne Aubert[a#]

Affiliations :

[a]Laboratoire de Chimie Bactérienne, UMR7283, Centre National de la Recherche Scientifique, Aix-Marseille Université, IM2B, IMM, Marseille, France.

[b]Institut Méditerranéen de Biodiversité et d'Ecologie Marine et Continentale (IMBE), UMR CNRS 7263, IRD 237, Aix Marseille Université, Avignon Université, Station Marine d'Endoume, Marseille, France.

[c]Aix Marseille Univ, CNRS, Centrale Marseille, iSm2, 13013 Marseille, France

Table S1

Features significantly more intense in 2PRIM cultivation conditions.

Structural Annotation based on MS2 spectra analysis and using *in silico* tools (SIRIUS, MetFrag)

**cluster IDX**[a] *(CPD IDX)*	**RT (min)**	**m/z**		**Ion type**	**Molecular formula**	**Natural Product Classifier**[b]		***In silico* scores**[c]		**Annotation (PCID)**[d]
		**Meas.**	**Calc**			**Pathway**	**Prob.**	**MetFrag**	**SIRIUS**	
93 *(39)*	2.16	258.2179	258.2181	$[M + H]^+$	$C_{13}H_{27}N_3O_2$	Alkaloids	0.99	ND	57.67	N1, N8-bis(ethyl)**spermidine**
112 *(4)*	2.72	286.1764	286.1766	$[M + H]^+$	$C_{13}H_{23}N_3O_4$	AA & peptides	0.99	ND	83.52	H-Gly-DL-Leu-DL-Pro-OH
209 *(4)*	5.08	286.1764	286.1767	$[M + H_3N + H]^+$	$C_{13}H_{20}N_2O_4$	AA & peptides	0.99	ND	79.51	deamino-Asp (pyrrol-1-yl)-Pro-OH
310 *(21)*	6.28	514.2701	514.2666	$[M + H]^+$	$C_{26}H_{35}N_5O_6$	AA and Peptides	0.99	ND	66.02	H-Gly-DL-Phe-DL-Lys-DL-Tyr-OH
318 *(54)*	6.37	321.1482	321.1484	$[M + H]^+$	$C_{13}H_{24}N_2O_5S$	*Fatty acids*	0.65	1	87.71	S-acetyl-pantethein
296 *(5)*	6.19	221.1264	221.1266	$[M + Na]^+$	$C_{10}H_{18}N_2O_2$	Alkaloids	0.65	ND	49	cyclo(valylvalyl)
518 *(5)*	8.63	175.0868	175.0871	$[M - H_2O + H]^+$	$C_{10}H_{12}N_2O_2$	Alkaloids	0.99	ND	71.19	3-[2-(hydroxyamino)ethyl]-1H-indol-5-ol
**582** ***(5)***	**9.53**	**521.2384**	**521.2400**	$[M + H]^+$	$C_{28}H_{32}N_4O_6$	**Alkaloids**	**0.95**	**ND**	**46.12**	**DKxanthene 520**
**628** ***(5)***	**10.75**	**543.26**	**543.2607**	$[M - H_2O + H]^+$	$C_{31}H_{36}N_4O_6$	**Alkaloids**	**0.57**	**ND**	**ND**	**DKxanthene 560 -H_2O**
475 *(16)*	8.17	657.3974	657.3977	$[M + H]^+$	$C_{34}H_{52}N_6O_7$	AA & peptides	0.99	ND	92.8	H-Val-Val-Val-Pro-Pro-Phe-OH
500 *(16)*	8.45	754.4493	754.4463	$[M + H]^+$	$C_{34}H_{59}N_9O_{10}$	AA & peptides	0.99	1	68.37	H-Leu-Gly-Ile-Pro-Gln-Asn-Leu-OH
708 *(30)*	14.86	649.4248	649.4315	$[M + H]^+$	$C_{37}H_{60}O_9$	Polyketides	0.93	1	50.74	macrocyclic lactone
715 *(30)*	15.12	658.4373	658.4388	$[M + H]^+$	$C_{35}H_{64}NO_8P$	Fatty acids	0.99	ND	87.02	PE 10:0_20:3
712 *(14)*	15.09	581.4779	581.4781	$[M + H]^+$	$C_{35}H_{64}O_6$	Fatty acids	0.98	ND	44.78	ND

cluster IDX[a] *(CPD IDX)*	RT (min)	m/z		Ion type	Molecular formula	Natural Product Classifier[b]		*In silico* scores[c]		Annotation (PCID)[d]
		Meas.	Calc			Pathway	Prob.	MetFrag	SIRIUS	
714 *(30)*	15.09	689.532	689.5332	[M + Na]$^+$	$C_{40}H_{74}O_7$	Fatty acids	0.90		48.64	DG derivative
713 *(14)*	15.09	667.5498	667.5512	[M + H]$^+$		Terpenoids	0.99	1	25.87	
755 *(14)*	16.69	649.5389	649.5407	[M + H]$^+$	$C_{40}H_{72}O_6$	Terpenoids	0.98	0.89	69.23	ND
754 *(14)*	16.69	631.5285	631.5301	[M - H_2O + H]$^+$		Fatty acids	0.99	1	96.8	ND
759 *(14)*	16.79	545.4552	545.4569	[M - H_2O + H]+	$C_{35}H_{62}O_5$	Terpenoids	0.99	1	49.22	ND
760 *(14)*	16.78	563.4663	563.4675	[M + H]$^+$		Terpenoids	0.99			
761 *(86)*	16.79	585.4469	585.4495	[M + Na]$^+$		*Terpenoids*	*0.40*			
764 *(14)*	16.83	651.5544	651.5563	[M + H]$^+$	$C_{40}H_{74}O_6$	Fatty acids	0.99	0.93	79.02	Erythritol 2,3 Dioleate
767 *(14)*	16.93	565.4818	565.4832	[M + H]$^+$	$C_{35}H_{64}O_5$	*Terpenoids*	*0.44*	0.93	87.84	DG 12:0_20:2
800 *(14)*	18.34	511.4507	511.4515	[M - H_2O + H]$^+$	$C_{35}H_{60}O_3$	Terpenoids	0.98	0.92	64.86	ND
813 *(14)*	19.47	523.4513	523.4515	[M - H_2O + H]$^+$	$C_{36}H_{60}O_3$	Terpenoids	0.99	1	83.09	Triterpenoid
814 *(14)*	19.47	541.461	541.4620	[M + H]$^+$		Terpenoids	0.99	0.96	79.59	
816 *(14)*	19.56	525.4668	525.4672	[M - 2H_2O + H]+	$C_{36}H_{64}O_4$	Terpenoids	0.76	0.96	47.50	Prenylated steroid
817 *(14)*	19.56	543.477	543.4777	[M - H_2O + H]+		Terpenoids	0.76	1	75.47	
806 *(26)*	19.28	591.5127	591.5141	[M - H_4O_2 + H]$^+$	$C_{41}H_{70}O_4$	*Fatty acids*	*0.25*	0.99	45.19	DG 16:0_22:6
807 *(26)*	19.28	609.5226	609.5246	[M - H_4O_2 + H]$^+$	$C_{41}H_{72}O_5$	Fatty acids	0.99	0.99	89.69	DG 18:2_20:2
808 *(26)*	19.28	627.5338	627.5352	[M - H_2O + H]$^+$	$C_{41}H_{72}O_5$	Fatty acids	0.99	1	95.48	DG 16:2_22:2 or DG 16:0_22:4
789 *(86)*	18.01	599.4589	599.4620	[M + Na]$^+$	$C_{36}H_{64}O_5$	Fatty acids	0.90	1	47.48	DG 18:3_15:0

[a]The Cluster index (IDX) identifies nodes, while the component index (CPD IDX) identifies spectral family, in the GNPS molecular network (job ID: https://gnps.ucsd.edu/ProteoSAFe/status.jsp?task=cd2673eb70ea47aa9561d6902ec36aee). [b]Natural product classifier within SIRIUS was used to propose structural class associated with its probability (Prob) score for each annotated feature. In red structural class with a low probability score [c]SIRIUS Tanimoto score is given when a putative ID is proposed, with the confidence levels 3 (Schymanski et al. 2014). [d]PCID = Pubchem ID number of the corresponding proposed structure. ND: Not determined mainly for compound with molecular formula but without putative structure (confidence level 4). Noisy Spectra preventing accurate determination of molecular formula and in-source fragments were not represented in the table.

- **Out of the 41 MS features a total of 24 molecules were putatively identified following manual curation of MS2 spectra**

REFERENCE:

Schymanski, Emma L., Junho Jeon, Rebekka Gulde, Kathrin Fenner, Matthias Ruff, Heinz P. Singer, and Juliane Hollender. "Identifying Small Molecules via High Resolution Mass Spectrometry: Communicating Confidence." *Environmental Science & Technology* 48, no. 4 (February 18, 2014): 2097–98. https://doi.org/10.1021/es5002105.

.

Table S2
ESI-MS^2 data of S-acyl-pantetheine derivatives (spectral family 54)

Cluster IDX[a]	RT (min)	*m/z* Meas.	*m/z* Calc.	error (ppm)	ion type	ion formula	MS$^{2\ b}$ *m/z* Frag.	MS$^{2\ b}$ Int.(%)	Proposed structure ID *NAME & SMILE*	*PCID*[c]
318	6.38	321.1479	321.1480	-0.5	$[M + H]^+$	$C_{13}H_{25}N_2O_5S$	103.0217	26.4	**S-acetyl-pantetheine** CC(=O)SCCNC(=O)CCNC(=O)C(C(C)(C)CO)O	101639605
							114.0553	40.0		
							120.0482	29.3		
							132.0483	14.3		
							149.0746	25.0		
							191.0852	100		
							303.1374	22.9		
396	7.27	335.1635	335.1639	-1.3	$[M + H]^+$	$C_{14}H_{27}N_2O_5S$	117.0372	10.0	**S-propionyl-pantetheine** CCC(=O)SCCNC(=O)CCNC(=O)C(C(C)(C)CO)O	146597969
							128.0708	77.0		
							132.0480	17.0		
							149.0745	34.0		
							205.1008	100		
							317.1533	31.0		
469	8.07	349.1792	349.1788	0.9	$[M + H]^+$	$C_{15}H_{29}N_2O_5S$	100.0759	9.9	**S-butyryl-pantetheine** CCCC(=O)SCCNC(=O)CCNC(=O)C(C(C)(C)CO)O	88197339
							132.0479	15.1		
							142.0863	100		
							149.0743	23.7		
							219.1163	95.7		
							331.1689	30.1		
532	8.85	363.1948	363.1942	1.6	$[M + H]^+$	$C_{16}H_{31}N_2O_5S$	85.0650	14.0	**S-isovaleryl-pantetheine** CC(C)(CO)C(O)C(=O)NCCC(=O)NCCSC(=O)CC(C)C	/
							102.0376	15.3		
							114.0916	11.6		
							132.0479	24.6		
							149.0744	66.7		
							156.1020	98.2		
							233.1322	100		
							345.1847	26.3		

[a]The Cluster index (IDX) identifies nodes in the GNPS molecular network (job ID: cd2673eb70ea47aa9561d6902ec36aee). [b]Major MS² fragments and their intensities were selected using the GNPS Metabolomics USI tool. [c]PCID = Pubchem ID number of the corresponding proposed structure. The putative structure identities are given with the confidence level 2-2b according to Schymanski et al. 2014.

Figure S1

Annotated MS2 spectra of S-acyl-pantetheine derivatives clustering with S-acetyl-pantetheine

spectral family 54- GNPS JOB ID: d2673eb70ea47aa9561d6902ec36aee

FT396 (Cluster index 396), identified as S-propionyl-pantetheine (confidence level 2)

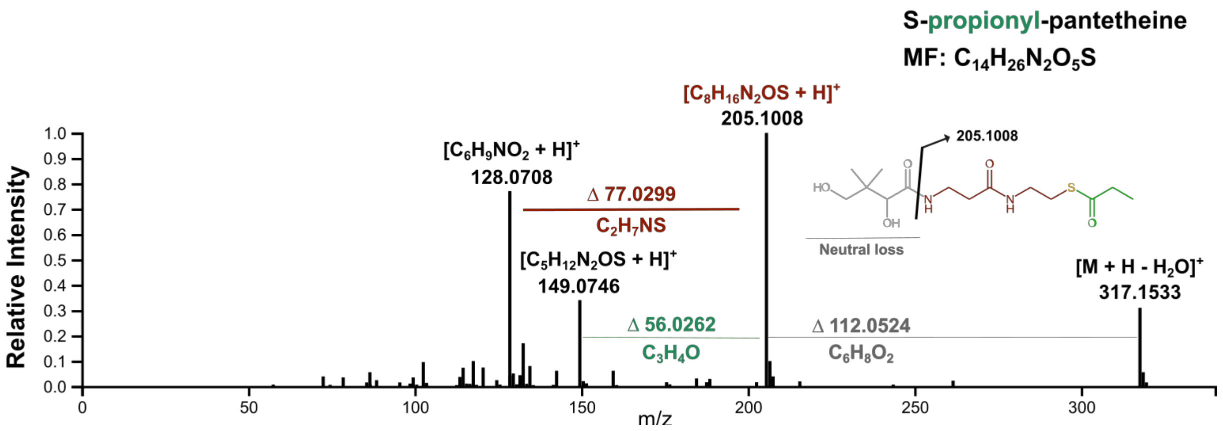

FT469 (Cluster index 469), identified as S-butyryl-pantetheine (confidence level 2)

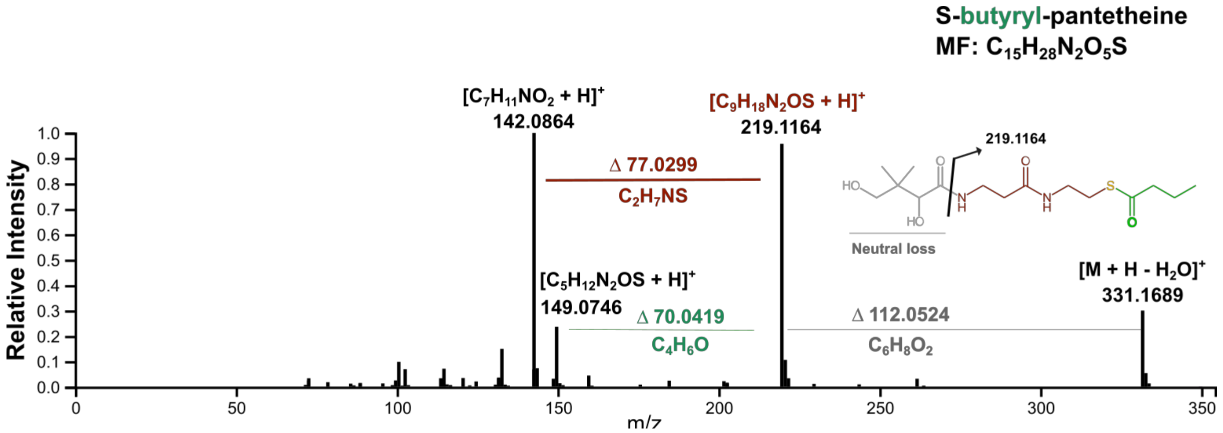

FT532 (Cluster index 532), identified as S-isovaleryl-pantetheine (confidence level 2)

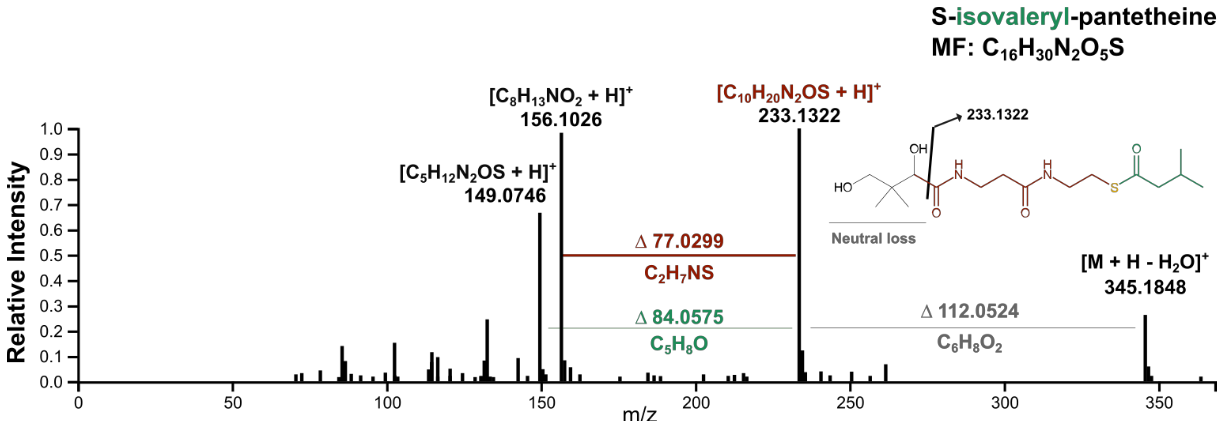

Table S3
ESI-MS^2 data of dereplicated *M. xanthus* specialized metabolites

Common Name	Cluster IDX[a] (CPD IDX)	RT (min)	meas. *m/z*	calc. *m/z*	error (ppm)	ion type	MS^{2} [b] *m/z* frag.	MS^{2} [b] Int.(%)	Molecular Formula	PubChem ID	CL[c]
**Myxoprin. C-506**	**329 (21)**	6.47	506.2731	506.2715	-3.2	$[M + 2H]^{2+}$	797.4018	2.70	$C_{45}H_{74}N_{10}O_{16}$	101561351	2
							682.3393	2.2			
							437.2035	3.40			
							330.2029	6.90			
							187.1444	100			
**Myxochelin B**	**375 (-1)**	6.99	404.1796	404.1816	5	$[M + H]^{+}$	387.1554	39.10	$C_{20}H_{25}N_3O_6$	10873133	2
							268.1656	11.60			
							251.1395	37.50			
							137.0233	93.8			
							98.0966	100			
**Cittilin B**	**425 (89)**	7.65	617.2609	617.2606	-0.5	$[M + H]^{+}$	589.2648	19.80	$C_{33}H_{36}N_4O_8$	101760076	2
							504.1755	38.1			
							476.1820	40.5			
							448.1874	100			
							213.1245	11.4			
**Cittilin A**	**445 (89)**	7.89	631.2774	631.2762	-1.8	$[M + H]^{+}$	603.2808	18.6	$C_{34}H_{38}N_4O_8$	101859253	2
							518.1929	28.3			
							490.1977	26.9			
							462.2028	100			
							445.1754	8.0			
**Myxochelin A**	**505 (-1)**	8.45	405.1634	405.1656	5.4	$[M + H]^{+}$	387.155	17.80	$C_{20}H_{24}N_2O_7$	16093504	2
							269.1478	35.00			
							251.1388	15.00			
							170.0602	66.7			
							137.0231	100			

Common Name	Cluster IDX[a] (CPD IDX)	RT (min)	meas.	calc.	error (ppm)	ion type	MS[2 b]		Molecular Formula	PubChem ID	CL[c]
			m/z				***m/z* frag.**	**Int.(%)**			
**DKxanthene-534**	**587 (5)**	9.60	535.2553	535.2551	-0.4	[M + H]+	416.2182	2.2	$C_{29}H_{34}N_4O_6$	101859252	**2**
							399.1917	4.3			
							268.1700	9.5			
							251.1432	7.8			
							120.0447	100			
**DKxanthene-518**	**598 (5)**	9.87	519.2600	519.2602	0.5	[M + H]+	383.1972	2.90	$C_{29}H_{34}N_4O_5$	139583444	2
							268.1707	10.60			
							251.1437	8.60			
							143.0858	9.8			
							120.0448	100			
**DKxanthene-560**	**610 (5)**	10.22	561.2711	561.2708	-0.7	[M + H]+	442.2340	1.8	$C_{31}H_{36}N_4O_6$	139588508	2
							425.2077	4.9			
							294.1860	6.20			
							277.1591	7.1			
							120.0447	100			
**DKxanthene-544**	**624 (5)**	10.57	545.2778	545.2758	-3.5	[M + H]+	426.2404	1.3	$C_{31}H_{36}N_4O_5$	139585142	**2**
							409.2131	2.7			
							294.1851	6.5			
							277.1598	8			
							120.0448	100			
**Myxalamid C**	**711 (10)**	14.92	388.2846	388.2846	0.1	[M + H]+	290.2105	17.5	$C_{24}H_{37}NO_3$	11954001	2
							215.1429	79.2			
							187.1480	58.3			
							107.0857	95.8			
							99.0805	100			
**Myxalamid B**	**726 (10)**	15.52	402.3004	402.3003	-0.4	[M + H]+	384.2896	3.80	$C_{25}H_{39}NO_3$	5282085	2
							290.2115	13.3			

Common Name	Cluster IDX[a] (CPD IDX)	RT (min)	meas.	calc.	error (ppm)	ion type	MS[2 b]		Molecular Formula	PubChem ID	CL[c]
			m/z				***m/z* frag.**	**Int.(%)**			
							215.1432	51.1			
							187.1482	26.1			
							95.0858	100			
**Myxovirescin A**	**732 (-1)**	15.80	624.4459	624.447	1.7	$[M + H]^+$	574.4113	100.00	$C_{35}H_{61}NO_8$	6450484	2
							538.3926	44.80			
							439.3217	37.90			
							270.1705	41.4			
							225.1643	79.3			
**Myxalamid A**	**738 (10)**	16.03	416.3163	416.3159	-0.9	$[M + H]^+$	398.3052	8.8	$C_{26}H_{41}NO_3$	6440913	2
							290.2115	25.0			
							215.1432	90.4			
							187.1483	42.3			
							109.1015	100			

[a]The Cluster index identifies nodes in the GNPS molecular network. [b]Major MS² fragments and their intensities were selected using the GNPS Metabolomics USI tool. [c]Confidence level of metabolite identification according to Schymanski et al. (2014) *N = noisy spectra*
Corresponding GNPS job: https://gnps.ucsd.edu/ProteoSAFe/status.jsp?task=cd2673eb70ea47aa9561d6902ec36aee

REFERENCE:

Schymanski, Emma L., Junho Jeon, Rebekka Gulde, Kathrin Fenner, Matthias Ruff, Heinz P. Singer, and Juliane Hollender. "Identifying Small Molecules via High Resolution Mass Spectrometry: Communicating Confidence." *Environmental Science & Technology* 48, no. 4 (February 18, 2014): 2097–98. https://doi.org/10.1021/es5002105.

Figure S2

Annotated MS^2 spectra (ESI +, CE:20-40eV) of dereplicated *M. xanthus* metabolites

Myxoprincomide C-506 (cluster index 319)

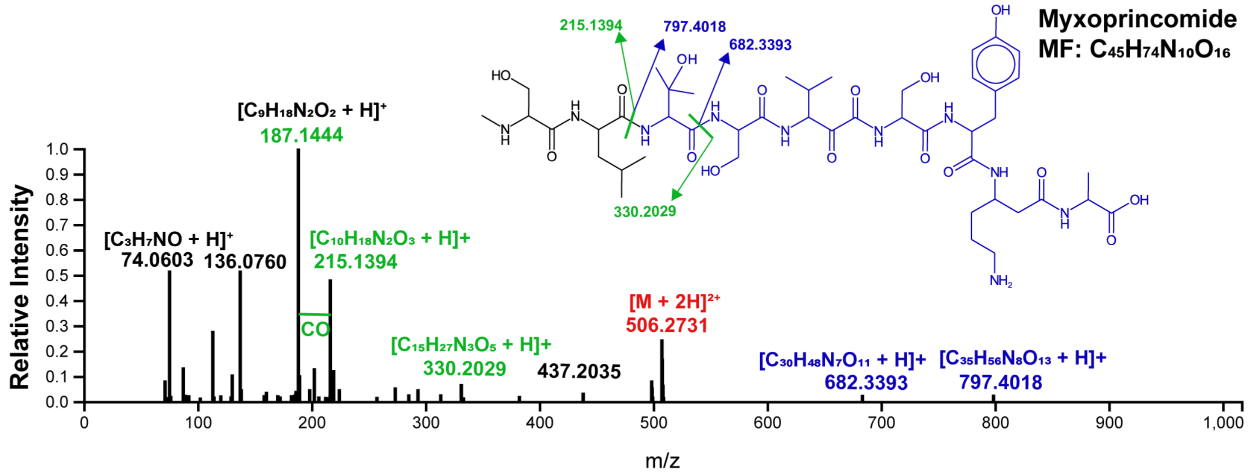

Myxochelin B (cluster index 375)

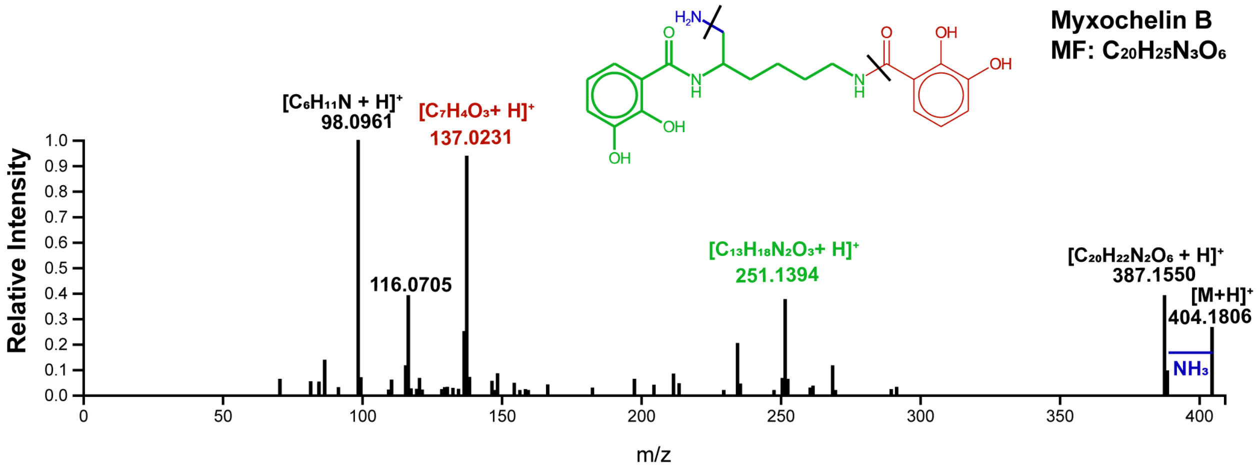

Myxochelin A (cluster index 505)

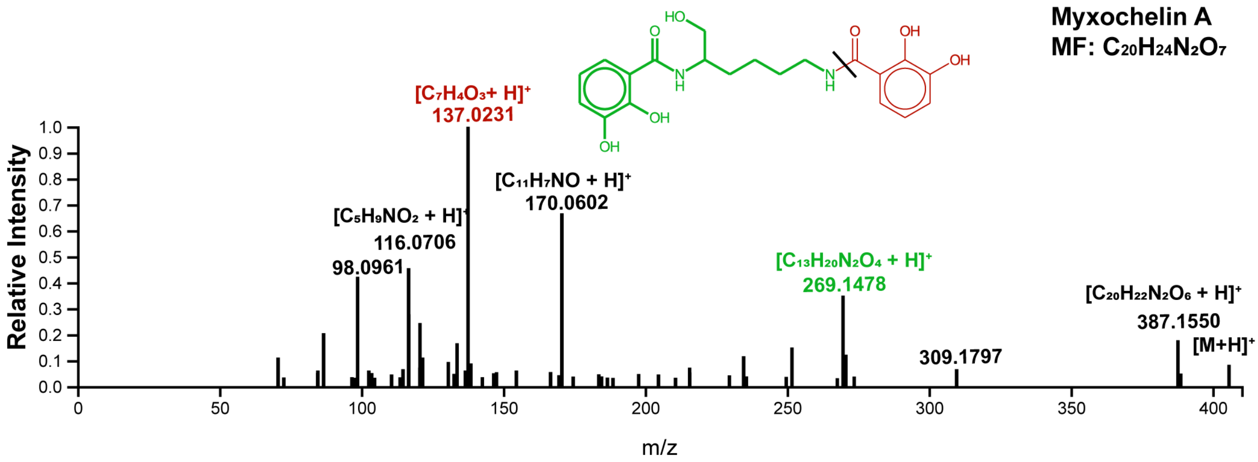

DKxanthene 534 (cluster index 587)

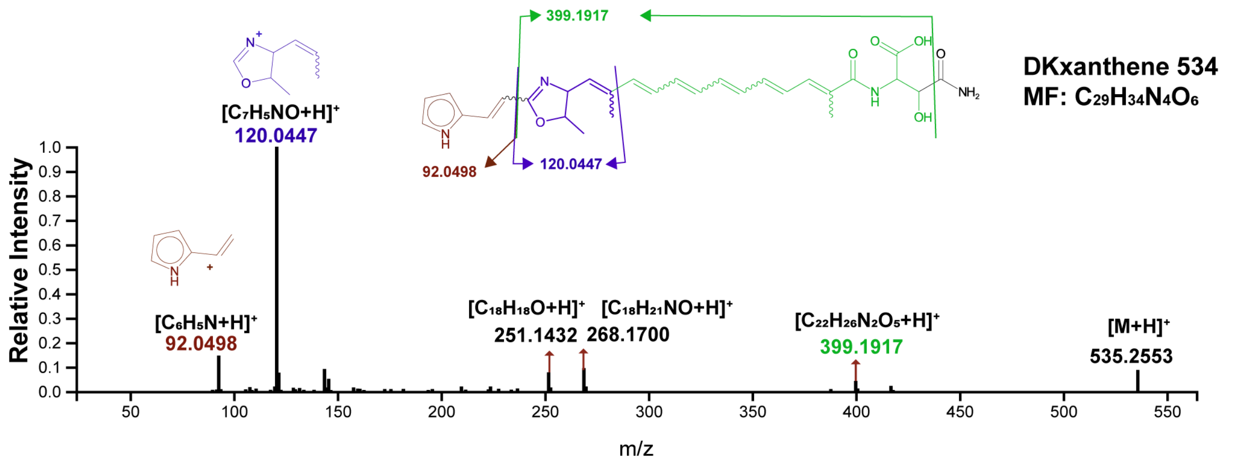

Myxalamid A (cluster index 738)

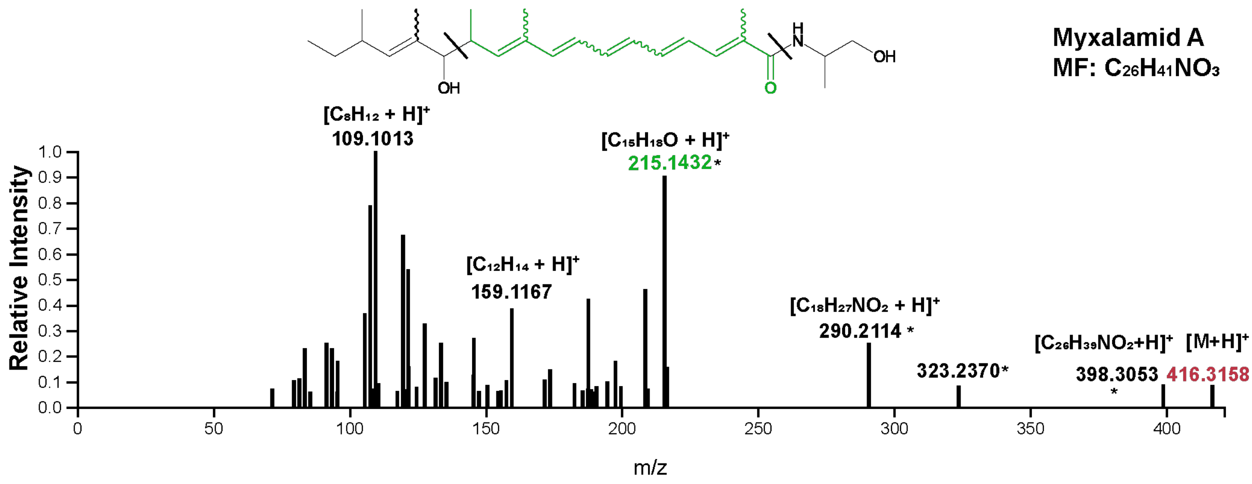

Cittilin A (cluster index 445)

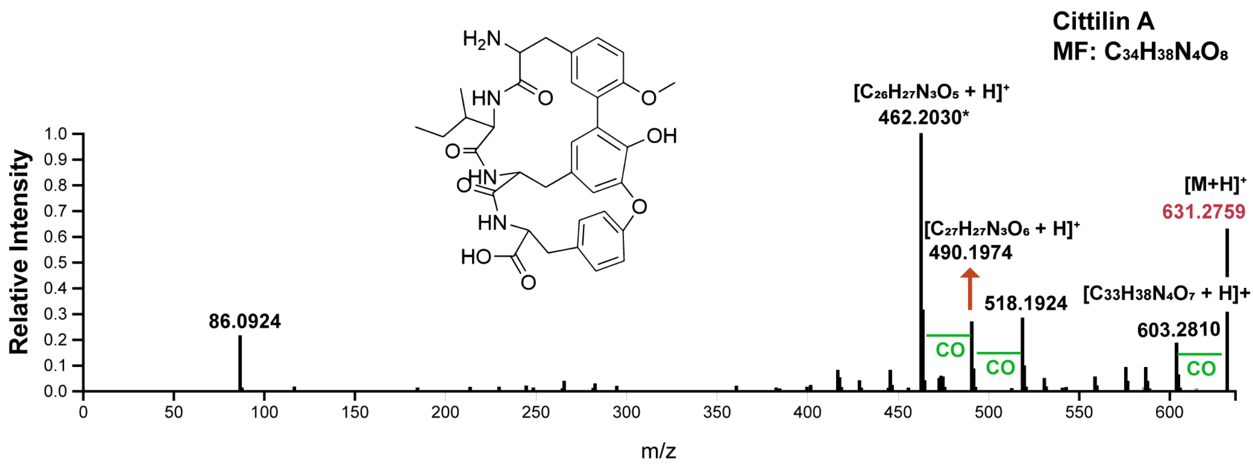

Myxovirescin A (cluster index 732)

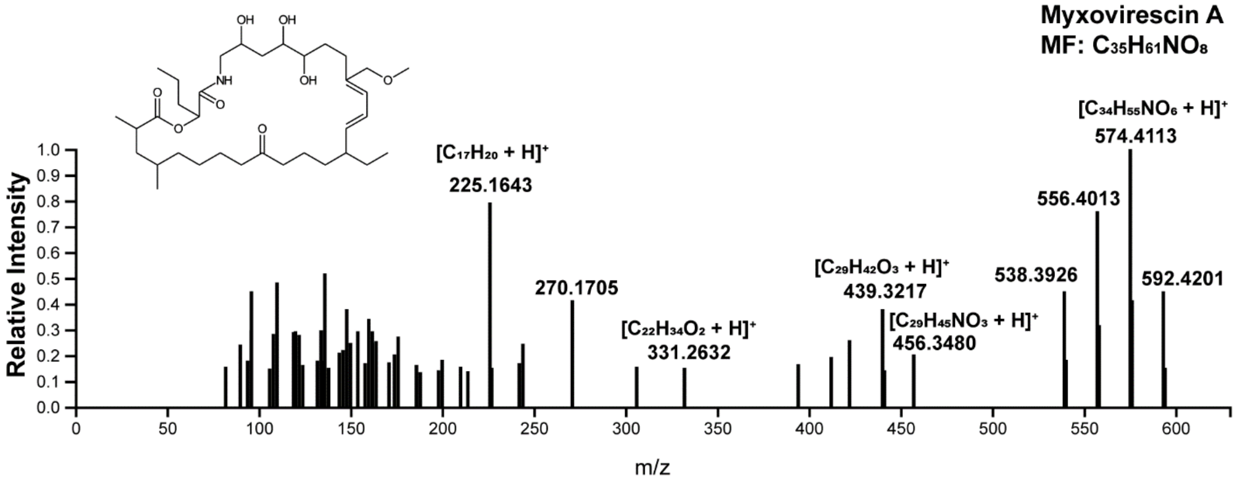

Corresponding GNPS job:
https://gnps.ucsd.edu/ProteoSAFe/status.jsp?task=cd2673eb70ea47aa9561d6902ec36aee

Figure S3

Predation of *E. coli* by *M. xanthus* on minimal medium

On the left *M. xanthus* colony that predates *E. coli* colony on the right. The predation of the BOOST strain and the simple mutants of MXC- = myxochelins null strain, DKx- = DKxanthenes null strain, MXV- = myxovirescin null strain and MXA- = myxalamids null strain are compared with the predation of the WT. Pictures of the predation were taken after 72 h of growth on CF plates with 1,5 % agar.

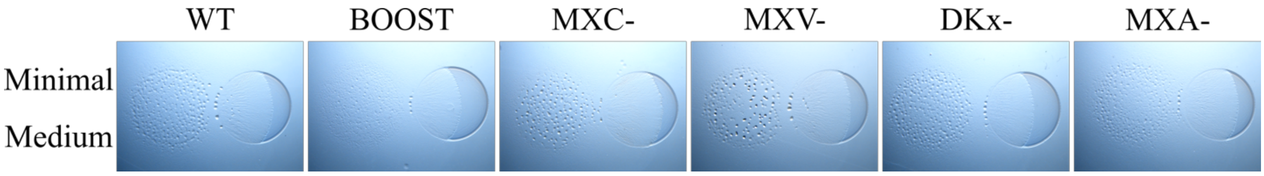

Table S4
Features significantly more intense in extracts from BOOST strain cultivated in 2PRIM conditions.

Cluster IDX[a] (CPD IDX)	RT (min)	*m/z* Meas.	*m/z* Calc.	error (ppm)	Ion status	Ion formula	MS[2.b] *m/z* frag.	MS[2.b] Int.(%)	Putative ID	SIRIUS[c] SCORE	PubChem ID
44 (41)	1.39	217.1544	217.1547	1	$[M+H]^+$	$C_{10}H_{21}N_2O_3$	72.0806	61.8	Valylvaline	79.61	CNP0082603
							112.0757	100			
							130.0867	42.7			
							140.1069	48.2			
							200.1287	13.6			
54 (-1)	1.72	173.1647	173.1648	0.5	$[M+H]^+$	$C_9H_{21}N_2O$	86.0965	100	N-[4-(propylamino) butyl] acetamide	55.93	14484863
							103.1227	7.50			
							133.0519	8.00			
							148.9766	6.00			
							156.1385	21.0			
92 (15)	3.92	312.1916	312.1918	0.6	$[M+H]^+$	$C_{15}H_{26}N_3O_4$	70.0649	27.8	Val-Pro-Pro	91.76	133109161
							116.0706	42.2			
							169.1335	100			
							197.1284	37.8			
							215.1400	6.7			
98* (-1)	4.34	479.1886	479.1887	0.3	$[M+2H]^{2+}$	$C_{40}H_{54}N_{12}O_{16}$	138.0548	100	ND	ND	ND
							168.0654	24.6			
							186.0759	26.2			
							204.0864	31.5			
							276.1079	18.5			
133* (36)	5.94	439.0340	439.0306	7.74	$[M + K]^+$	$C_{19}H_{14}NO_9$	136.0615	100	Polyaromatic structure	ND	ND
							159.0582	6.70			
							213.1228	16.4			
							312.0490	4			
							412.0144	10.4			
173 (-1)	6.66	513.2791	513.2793	0.5	$[M+2H]^{2+}$	$C_{46}H_{78}N_{10}O_{16}$	***136.0750***	***29.5***	Methylated myxoprin-comide	ND	ND
							173.0913	45.9			
							187.1430	***41.0***			
							270.1449	100			
							330.2006	***3.60***			

Cluster IDX[a] (CPD IDX)	RT (min)	*m/z*		error (ppm)	Ion status	Ion formula	MS[2.b]		Putative ID	SIRIUS[c] SCORE	PubChem ID
		**Meas.**	**Calc.**				***m/z* frag.**	**Int.(%)**			
316 (30)	13.59	452.2746	452.2772	5.6	$[M+H]^+$	$C_{21}H_{43}NO_7P$	89.0596	7.60	PE (16:1(9Z)/0:0)	99.22	134727885
							121.1008	4.80			
							133.0856	6.00			
							280.2630	3.80			
							311.2578	100			
341 (30)	15.42	468.3076	468.3085	1.9	$[M+H]^+$	$C_{22}H_{47}NO_7P$	89.0590	23.7	PE (17:0/0:0)	100	86583376
							109.1005	11.1			
							133.0856	11.6			
							296.2957	8.9			
							327.2885	100			
435 (-1)	22.34	570.548	570.5456	-4.2	$[M+H]^+$	$C_{35}H_{72}NO_4$	254.2846	14.5	Cer 21:0;2O/14:0;(3OH)	92.09	138161835
							266.2844	87.2			
							284.2949	100			
							534.5261	7.9			
							552.5361	57.4			

[a]The Cluster index (IDX) identifies nodes, while the component index (CPD IDX) identifies spectral family, in the GNPS molecular network (job ID: 9a7b4f773d734045ae880cb5ebdd34e6). [b]Major MS^2 fragments and their intensities were selected using the GNPS Metabolomics USI tool. [c]SIRIUS Tanimoto score for the proposed putative structure, whose identity is given with the confidence level 3-4. * features annotated with a confidence level 4 (Schymanski et al. 2014). ND: Not Determined.

REFERENCE:

Schymanski, Emma L., Junho Jeon, Rebekka Gulde, Kathrin Fenner, Matthias Ruff, Heinz P. Singer, and Juliane Hollender. “Identifying Small Molecules via High Resolution Mass Spectrometry: Communicating Confidence.” *Environmental Science & Technology* 48, no. 4 (February 18, 2014): 2097–98. https://doi.org/10.1021/es5002105.

Figure S4
Calibration curves from integrated area for EIC corresponding to Myxoprincomide c-506.

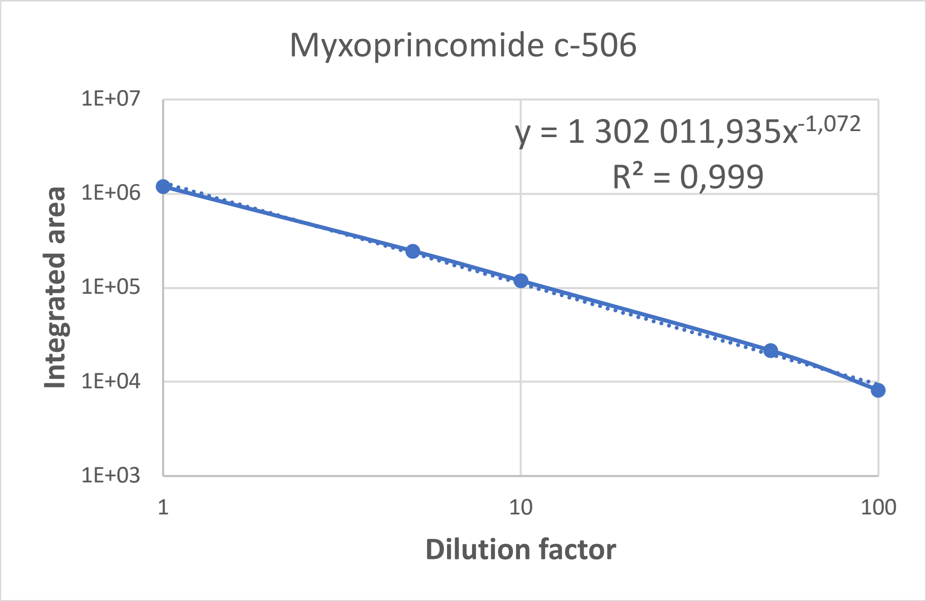

Table S5

Myxoprincomide c-506 concentrations in 2PRIM protocol extracts.

Areas under the curve of each extracted ion chromatograms of myxoprincomide c-506 were reported to the calibration curve Figure S4. Data were first normalized to the concentration of myxoprincomide of WT 2PRIM protocol extracts and then normalized a second time to the concentration of myxoprincomide of WT standard protocol extracts.

Sample	Area under the curve	Reported to calibration curve	Normalized to WT 2PRIM	Normalized to WT standard (x31)	Mean
WT	17404,16	55,99	0,59	18,31	31,00
WT	11309,78	83,70	0,88	27,38	
WT	8411,09	110,33	1,16	36,09	
WT	7110,05	129,05	1,36	42,22	
BOOST	14286,90	67,31	1,41	43,65	48,59
BOOST	15965,39	60,68	1,56	48,41	
BOOST	15089,87	63,96	1,48	45,93	
BOOST	18797,51	52,11	1,82	56,38	
BOOST_MXP	128806,06	8,65	10,95	339,49	389,58
BOOST_MXP	181293,70	6,29	15,06	466,98	
BOOST_MXP	158894,08	7,11	13,32	412,92	
BOOST_MXP	128585,81	8,67	10,93	338,94	

Figure S5
Molecular network of the BOOST_MXP strain in 2PRIM condition.

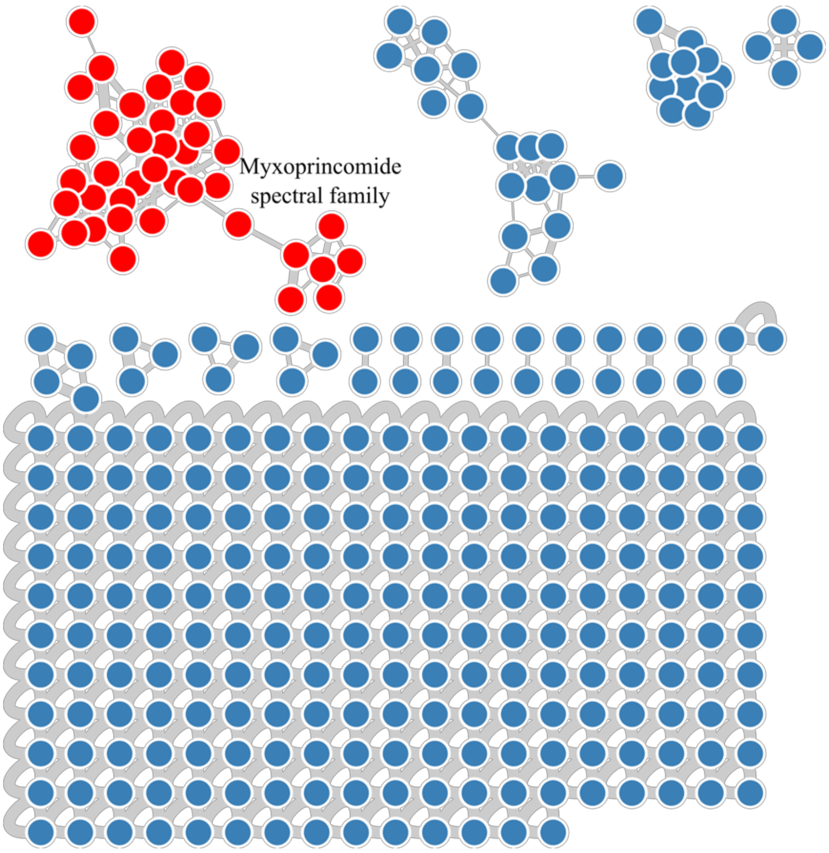

Table S6

Myxoprincomide C-506 congeneric metabolites (directly linked to C-506 within the spectral family 2 containing 40 features)

Cluster IDX[a]	RT (min)	*m/z*		error (ppm)	Ion status	Molecular Formula[a]	MS² spectra analysis[b]				Structure ID[c]
		meas.	Calc.				*m/z*	Int. (%)	Formula	Annotation	
251	6.03	507.2797	507.2793	0.3	$[M+2H]^{2+}$	$C_{45}H_{76}N_{10}O_{16}$	912.4902		$C_{41}H_{70}N_{9}O_{14}$	$[M$ - N-MeSer + $H]^+$	**C-507= dihydro-C-506**
							799.4178	2.75	$C_{35}H_{59}N_{8}O_{13}$	$[M$ - N-MeSerLeu + $H]^+$	
							684.3545	2.46	$C_{30}H_{50}N_{7}O_{11}$	$[M$ - N-MeSerLeuOxVal + $H]^+$	
							**330.2025**	**6.29**	$\mathbf{C_{15}H_{28}N_{3}O_{5}}$	**[N-MeSerLeuOxVal + H]⁺**	
							292.1662	6.4	$C_{15}H_{22}N_{3}O_{3}$	[N-MeSerLeu**OxVal** - $2H_2O$ + $H]^+$	
							**215.1393**	**46.91**	$\mathbf{C_{10}H_{19}N_{2}O_{3}}$	**[N-MeSerLeu + H]⁺**	
							**187.1443**	**100**	$\mathbf{C_{9}H_{19}N_{2}O_{2}}$	**[N-MeSerLeu - CO + H]⁺**	
							136.0759	49.16	$C_{8}H_{10}NO$		
							112.076	27.42	$C_{6}H_{10}NO$		
							74.0602	54.72	$C_{3}H_{8}NO$		
63	6.18	528.2851	528.2846	0.95	$[M+2H]^{2+}$	$C_{47}H_{78}N_{10}O_{17}$	841.4305	2.0	$C_{37}H_{61}N_{8}O_{14}$	$[M$ - N-MeSerLeu + $H]^+$	**c-528 = OxLeu replace OxVal**
							726.3751	2.5	$C_{32}H_{51}N_{7}O_{12}$	$[M$ - N-MeSerLeu**OxLeu** + $H]^+$	
							**330.2012**	**8.9**	$\mathbf{C_{15}H_{28}N_{3}O_{5}}$	**[N-MeSerLeuOxLeu + H]⁺**	
							**215.1390**	**57.1**	$\mathbf{C_{10}H_{19}N_{2}O_{3}}$	**[N-MeSerLeu + H]⁺**	
							**187.1443**	**100**	$\mathbf{C_{9}H_{19}N_{2}O_{2}}$	**[N-MeSerLeu - CO + H]⁺**	
							136.0759	49.16	$C_{8}H_{10}NO$		
							112.076	27.42	$C_{6}H_{10}NO$		
							74.0602	48.4	$C_{3}H_{8}NO$		
12	6.22	515.2507	515.2496	2.13	$[M+2H]^{2+}$	$C_{44}H_{72}N_{10}O_{16}\mathbf{S}$	928.4510	0.41	$C_{40}H_{66}N_{9}O_{14}S$	$[M$ - N-MeSer + $H]^+$	**c-515 = Met replace Leu**
							797.4041	4.4	$C_{35}H_{57}N_{8}O_{13}$	$[M$ - N-MeSer**Met** + $H]^+$	
							682.3398	3.5	$C_{30}H_{48}N_{7}O_{11}$	$[M$ - N-MeSer**Met**OxVal + $H]^+$	
							437.2027	6.8	$C_{20}H_{29}N_{4}O_{7}$	[Ser-Val-Unk-Ser-Tyr + $H]^+$	
							**348.1600**	**8.8**	$\mathbf{C_{14}H_{26}N_{3}O_{5}S}$	[N-MeSer**MetOxVal** + $H]^+$	
							**233.0958**	**48.8**	$\mathbf{C_{9}H_{17}N_{2}O_{3}S}$	[N-MeSer**Met** + $H]^+$	
							**205.1005**	**100**	$\mathbf{C_{8}H_{17}N_{2}O_{2}S}$	[N-MeSer**Met** - CO + $H]^+$	
							**157.0969**	**26.8**	$\mathbf{C_{7}H_{13}N_{2}O_{2}}$	**[N-MeSerMet- CO-CH₃S + H]⁺**	

Cluster IDX[a]	RT (min)	*m/z*		error (ppm)	Ion status	Molecular Formula[a]	MS² spectra analysis[b]				Structure ID[c]
		meas.	Calc.				*m/z*	Int. (%)	Formula	Annotation	
							136.0747	78.0	$C_8H_{10}NO$		
							112.0753	46.3	$C_6H_{10}NO$		
							74.0994	56.1	C_3H_8NO		
156	6.27	514.2699	514.2695	0.78	$[M+2H]^{2+}$	$C_{45}H_{74}N_{10}O_{17}$	437.2031	6.7	$C_{20}H_{29}N_4O_7$	$[Ser-Val-Unk-Ser-Tyr + H]^+$	**c-514 = Oxidized c-506**
							**330.2030**	**7.7**	$\mathbf{C_{15}H_{28}N_3O_5}$	**$[N-MeSerLeuOxVal + H]^+$**	
							**215.1389**	**53.3**	$\mathbf{C_{10}H_{19}N_2O_3}$	**$[N-MeSerLeu + H]^+$**	
							**187.1438**	**100**	$\mathbf{C_9H_{19}N_2O_2}$	**$[N-MeSerLeu - CO + H]^+$**	
							136.0756	76.7	$C_8H_{10}NO$		
							112.0757	43.3	$C_6H_{10}NO$		
							74.0599	56.7	C_3H_8NO		
165	6.46	515.2772	515.2773	-0.19	$[M+2H]^{2+}$	$C_{45}H_{76}N_{10}O_{17}$	797.4032	2.2	$C_{35}H_{57}N_8O_{13}$	$[M - N-MeSerLeu + H]^+$	**c-506 *in source* hydrate**
							682.3430	2.3	$C_{30}H_{48}N_7O_{11}$	$[M - N-MeSerLeuOxVal + H]^+$	
							437.2031	4.5	$C_{20}H_{29}N_4O_7$	$[Ser-Val-Unk-Ser-Tyr + H]^+$	
							**330.2020**	**6.6**	$\mathbf{C_{15}H_{28}N_3O_5}$	**$[N-MeSerLeuOxVal + H]^+$**	
							**215.1389**	**50.0**	$\mathbf{C_{10}H_{19}N_2O_3}$	**$[N-MeSerLeu + H]^+$**	
							**187.1438**	**100**	$\mathbf{C_9H_{19}N_2O_2}$	**$[N-MeSerLeu - CO + H]^+$**	
							136.0754	55.3	$C_8H_{10}NO$		
							112.0756	28.9	$C_6H_{10}NO$		
							74.0599	56.7	C_3H_8NO		
155	6.46	506.2724	506.2715	1.78	$[M+2H]^{2+}$	$C_{45}H_{74}N_{10}O_{16}$	910.4979	0.33	$C_{41}H_{68}N_9O_{14}$	$[M - N-MeSer + H]^+$	**c-506** (101561351)
							797.403	3.78	$C_{35}H_{57}N_8O_{13}$	$[M - N-MeSerLeu + H]^+$	
							682.3447	2.95	$C_{30}H_{48}N_7O_{11}$	$[M - N-MeSerLeuOxVal + H]^+$	
							437.2024	3.75	$C_{20}H_{29}N_4O_7$	$[Ser-Val-Unk-Ser-Tyr + H]^+$	
							**330.2035**	**7.21**	$\mathbf{C_{15}H_{28}N_3O_5}$	[N-MeSer**LeuOxVal** + H]⁺	
							**215.1396**	**49.29**	$\mathbf{C_{10}H_{19}N_2O_3}$	[N-MeSer**Leu** + H]⁺	
							**187.1446**	**100**	$\mathbf{C_9H_{19}N_2O_2}$	[N-MeSer**Leu** - CO + H]⁺	
							136.0759	53.59	$C_8H_{10}NO$		
							112.0762	28.23	$C_6H_{10}NO$		

Cluster IDX[a]	RT (min)	*m/z*		error (ppm)	Ion status	Molecular Formula[a]	MS^2 spectra analysis[b]				Structure ID[c]
		meas.	Calc.				*m/z*	Int. (%)	Formula	Annotation	
							74.0602	51.38	C_3H_8NO		
7	6.65	513.2800	513.2793	1.36	$[M+2H]^{2}$	$C_{46}H_{76}N_{10}O_{16}$	437.2048	4.5	$C_{20}H_{29}N_4O_7$	$[Ser-Val-Unk-Ser-Tyr + H]^+$	**c-513 Methylated C-506**
							**330.2031**	**5.9**	$C_{15}H_{28}N_3O_5$	**[N-MeSerLeuOxVal + H]$^+$**	
							**215.1393**	**49.4**	$C_{10}H_{19}N_2O_3$	**[N-MeSerLeu + H]$^+$**	
							**187.1440**	**100**	$C_9H_{19}N_2O_2$	**[N-MeSerLeu - CO + H]$^+$**	
							136.0756	58.8	$C_8H_{10}NO$		
							112.0758	39.4	$C_6H_{10}NO$		
							74.0599	50.6	C_3H_8NO		
306	6.69	498.2749	498.2740	1.80	$[M+2H]^{2+}$	$C_{45}H_{74}N_{10}O_{15}$	894.5081	*0.23*	$C_{41}H_{68}N_9O_{13}$	[M - N-MeSer + H]$^+$	c-498 = Val replace OxVal
							781.4033	2.71	$C_{35}H_{57}N_8O_{12}$	[M - N-MeSerLeu + H]$^+$	
							682.3382	2.90	$C_{30}H_{48}N_7O_{11}$	[M - N-MeSerLeuVal + H]$^+$	
							437.1994	*5.33*	$C_{20}H_{29}N_4O_7$	[Ser-Val-Unk-Ser-Tyr + H]$^+$	
							**314.2067**	**11.8**	$C_{15}H_{28}N_3O_4$	[N-MeSer**LeuVal** + H]$^+$	
							**215.1385**	**58**	$C_{10}H_{19}N_2O_3$	[N-MeSer**Leu** + H]$^+$	
							**187.1433**	**100**	$C_9H_{19}N_2O_2$	[N-MeSer**Leu** - CO + H]$^+$	
							136.075	49.16	$C_8H_{10}NO$		
							112.0752	27.42	$C_6H_{10}NO$		
							74.0593	54.72	C_3H_8NO		
114	7.07	562.8143	562.8145	-0.35	$[M+2H]^{2+}$	$C_{51}H_{85}N_{11}O_{17}$	437.2044	5.5	$C_{20}H_{29}N_4O_7$	[Ser-Val-Unk-Ser-Tyr + H]$^+$	c-562 = c-506 + **Leu**
							**330.2035**	**9.5**	$C_{15}H_{28}N_3O_5$	**[N-MeSerLeuOxVal + H]$^+$**	
							**215.1393**	**50.0**	$C_{10}H_{19}N_2O_3$	**[N-MeSerLeu + H]$^+$**	
							**187.1446**	**100**	$C_9H_{19}N_2O_2$	**[N-MeSerLeu - CO + H]$^+$**	
							136.0755	40.5	$C_8H_{10}NO$		
							112.0758	32.0	$C_6H_{10}NO$		
							74.0599	37.0	C_3H_8NO		

[a]The Cluster index (IDX) identifies nodes in the GNPS molecular network. [b]Major MS^2 fragments and their intensities were selected using the GNPS Metabolomics USI tool. [c]Putative molecular identifications are proposed with a confidence level 2-3 (Schymanski et al. 2014). (https://gnps.ucsd.edu/ProteoSAFe/status.jsp?task=c5126a425d894bec8bf94e7219a8c1f1).

Figure S6

Representative MS² spectra mirror image with Myxoprincomide C-506 (FT155)

Comparative MS² spectra of FT12 with FT155

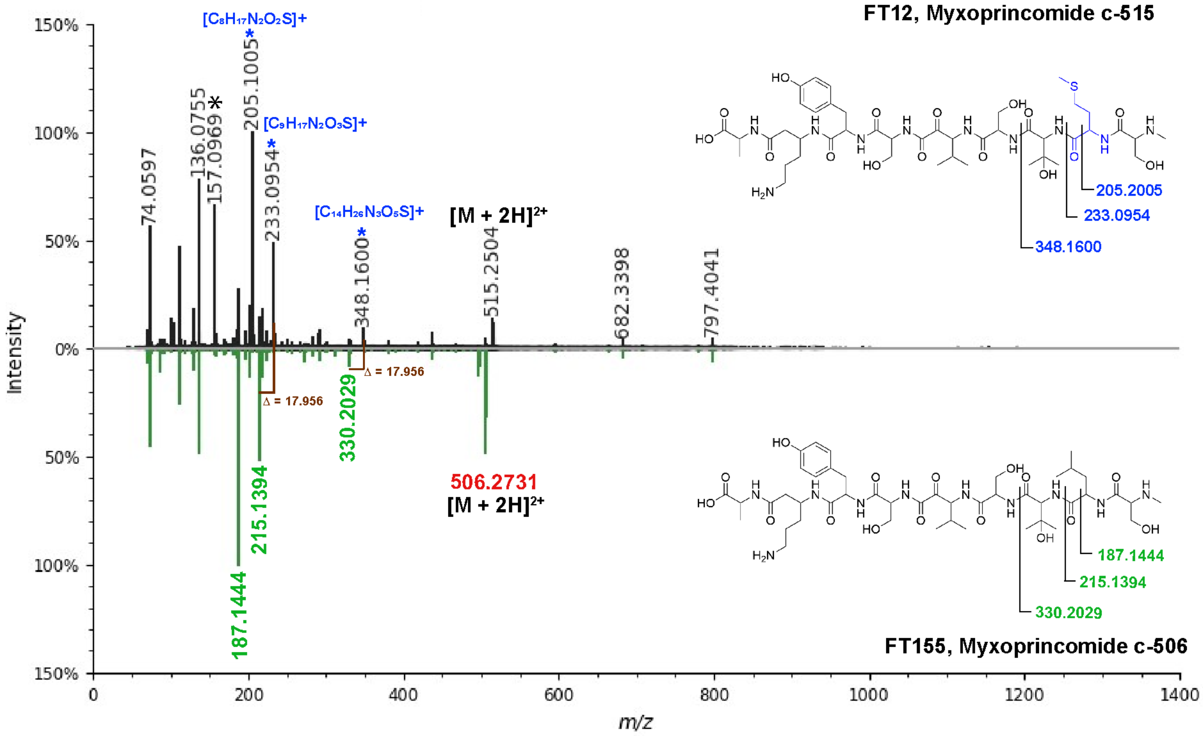

Comparative MS² spectra of FT306 with FT155

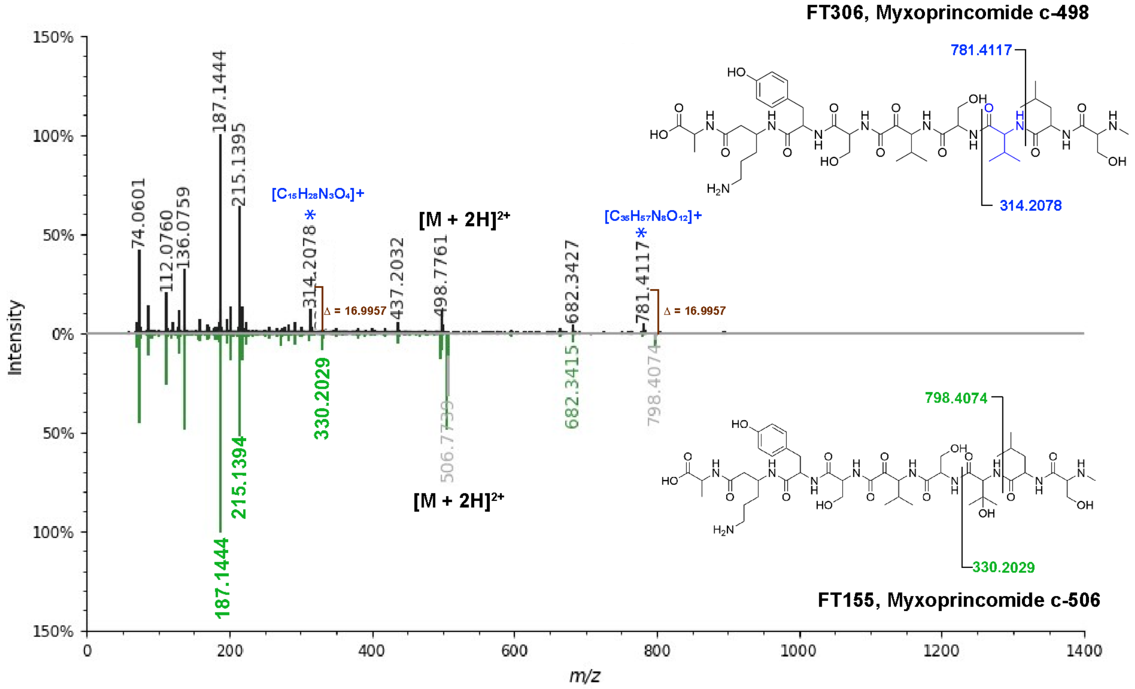

Table S7
Bacterial strains used in this study

**Strain**	**Relevant Genotype (comments)**	**Reference**
M. xanthus		
DZ2	Wild type strain	(1)
MXV-	DZ2 *Δmxan3936-3938* (Myxovirescin null)	This study
MXA-	DZ2 *Δmxan4530* (Myxalamids null)	This study
MXC	DZ2 *Δmxan3643* (Myxochelins null)	This study
DKx-	DZ2 *Δmxan4305* (DKxanthenes null)	This study
BOOST	DZ2 *Δmxan4305 Δmxan4530 Δmxan3643 Δmxan3936-3938*	This study
Δ2	DZ2 *Δmxan4305 Δmxan4530*	This study
Δ3	DZ2 *Δmxan4305 Δmxan4530 Δmxan3643*	This study
BOOST_MXP	DZ2 *Δmxan4305 Δmxan4530 Δmxan3643 Δmxan3936-3938 pBJ114-SP::mxan_3779*	This study
E. coli	*Escherichia coli* (ATCC 8739)	ATCC
B. subtilis	*Bacillus subtilis* (ATCC 6633)	ATCC
P. aeruginosa	*Pseudomonas aeruginosa* (ATCC 9027)	ATCC
K. pneumoniae	*Klebsiella pneumoniae* (DSM 26371)	DSMZ
S. aureus	*Staphylococcus aureus* (ATCC 6538P)	ATCC
A. baumanii	*Acinetobacter baumannii* (DSM 30007)	DSMZ
E. faecalis	Enterococcus faecalis DSM 2570	DSMZ
E. cloacae	Enterobacter cloacae subsp. Cloacae DSM 30054	DSMZ
C. albicans	*Candida albicans* (DSM 10697)	DSMZ
Eukaryotic cells		
A549	Cancer human lung epithelial cells A549 CCL-185 ™	ATCC

(1) Campos JM, Zusman DR. 1975. Regulation of development in *Myxococcus xanthus*: effect of 3':5'-cyclic AMP, ADP, and nutrition. Proc Natl Acad Sci U S A 72:518–522.

Table S8
Plasmids used in this study

**Plasmid**	**Relevant Genotype**	**Reference**
pBJ114	Used to create deletions or insertions, *galK,* KanR	Laboratory collection
pBJ114-*Δmxan3936-3938*	pBJ114 with deletion cassette for *mxan_3936 to mxan_3938*	This study
pBJ114-*Δmxan4530*	pBJ114 with deletion cassette for *mxan_4530*	This study
pBJ114-*Δmxan3643*	pBJ114 with deletion cassette for *mxan_3643*	This study
pBJ114-*Δmxan4305*	pBJ114 with deletion cassette for *mxan_4305*	This study
pBJ114_SP	pBJ114 with the J23104 Strong Promoter and BBa_B0034 RBS	This study
pBJ114-SP::*mxan_3779*	pBJ114 with insertion cassette for the creation of *SP*::*mxan_3779*	This study

Table S9

Primers used in this study

Primer	Sequence (5'-3')	Used for
114_hindIII_up3938_for	AACAGCTATGACATGATTACAAGCTTGGAGACAG GCCGACTCCCATGGGAAT	pBJ114-*Δmxan3936-3938*
up_down_3938-6_rev	GCGGCCAGAACCTCACGCGTTGTCATAGGGGCG CGCCTCAGAGGAACAGCTG	
up_down_3938-6_for	CAGCTGTTCCTCTGAGGCGCGCCCCTATGACAAC GCGTGAGGTTCTGGCCGC	
114_XbaI_down3936_rev	AGCTCGGTACCCGGGGATCCTCTAGAGTGCCCG AGCGAACTCGCGCAGTCGC	
MXAN_3938_for	TCACGCCCGTGGTCCTGGCCTG	Verification of *mxan3936-3938* deletion
MXAN_3938_rev	TCGATGAGACGGTCCGGCCGCG	
114_hindIII_up4530_for	AACAGCTATGACATGATTACAAGCTTAAAAACGA GCTCGGGTCGTCACCTGG	pBJ114-*Δmxan4530*
up_down_4530_rev	GCAATCTTCTCCGCGAACGTCGTCATTCCAAACG GAGCACCCCATGTCTCTC	
up_down_4530_for	GAGAGACATGGGGTGCTCCGTTTGGAATGACGA CGTTCGCGGAGAAGATTGC	
114_XbaI_down4530_rev	AGCTCGGTACCCGGGGATCCTCTAGAGAAGCCG TTGGCGCGGGCATCGAAGG	
MXAN_4530_for	GTGTCCGGTTCTCGTGAA	Verification of *mxan4530* deletion
MXAN_4530_rev	GCCGCGCACGAAGCCGTT	
114_hindIII_up3643_for	AACAGCTATGACATGATTACAAGCTTTCACGCAG GGCCAGTCGCCCGTGACG	pBJ114-*Δmxan3643*
up_down_3643_rev	TCTGGCGTCCAGGTGGGGTTCGTCACGCGTCAG GGGCGCGAGGTGACTCGCG	
up_down_3643_for	CGCGAGTCACCTCGCGCCCCTGACGCGTGACGA ACCCCACCTGGACGCCAGA	
114_XbaI_down3643_rev	AGCTCGGTACCCGGGGATCCTCTAGAGTCGAGCT GGCGGGTGCGCTCGCCAA	
MXAN_3643_for	ATGAACACACGGGAGTCC	Verification of *mxan3643* deletion
MXAN_3643_rev	AGGTGCCGCGCGCACGGG	
114_hindIII_up4305_for	AACAGCTATGACATGATTACAAGCTTCGCCTGTG CTCGGGGGAATGGGTGGG	pBJ114-*Δmxan4305*
up_down_4305_rev	AGGCTCCCTGGCGGCCCGTCACCGGCTCATAGGC CGAGCTGGTTGGCGATGA	
up_down_4305_for	TCATCGCCAACCAGCTCGGCCTATGAGCCGGTGA CGGGCCGCCAGGGAGCCT	

114_XbaI_down4305_rev	AGCTCGGTACCCGGGGATCCTCTAGAGACCTTCC ATTCCCCGTCGTGTTGCT	
MXAN_4305_for	ATGACACTCGACCAAATCGT	Verification of *mxan4305* deletion
MXAN_4305_rev	TCAAGGCGCCTGCGTCAG	
Int_SP_pbj114_For	GGAATTGTGAGCGGATAACAATTCCTCTAGAGAA AGAGGAGAAACTCGAGTACCGAGCTCGAATTCG GCACTGGCCGTC	pBJ114_SP
Int_SP_pbj114_Rev	TCCGCTCACAATTCCGCTAGCACAATACCTAGGA CTGAGCTAGCTGTCAACCCGGGGATCCTCTAGAG TCGACCTGCAG	
MXAN_3779_1000pbATG_for	AGAAACTCGAGATGCACGAGACTCCGCGAACCC	pBJ114-SP::*mxan_3779*
MXAN_3779_1000pbATG_rev	GTGCCGAATTCTGAGCGTGCCTTCGGCCAGGCC	
78_for	AGCGGATAACAATTTCACACAGGA	Verification of pBJ114-SP::*mxan_3779* integration

Table S10

Table of metadata for MS- based metabolomic analysis and feature based molecular networking on GNPS

ID	Strain	Protocol	Extract name	Attribute group	GNPS link
823	WT DZ2	Standard	WT_CTT-1_5mg.ml_(01-12-22)_BB5_1_6498.mzXML	WT_CTT	https://gnps.ucsd.edu/ProteoSAFe/status.jsp?task=cd2673eb70ea47aa9561d6902ec36aee
			WT_CTT-3_5mg.ml_(01-12-22)_BC1_1_6504.mzXML		
			WT_CTT-4_5mg.ml_(01-12-22)_BC5_1_6514.mzXML		
			WT_CTT-6_5mg.ml_(01-12-22)_BD1_1_6520.mzXML		
		2PRIM	WT_CF-1_5mg.ml_(23-11-22)_BB3_1_6496.mzXML	WT_CF	
			WT_CF-2_5mg.ml_(23-11-22)_BB8_1_6503.mzXML		
			WT_CF-3_5mg.ml_(23-11-22)_BC7_1_6516.mzXML		
			WT_CF-5_5mg.ml_(23-11-22)_RE4_1_6523.mzXML		
435	WT DZ2	2PRIM	WT_CF-1_5mg.ml_(23-11-22)_BB3_1_6496.mzXML	WT_CF	https://gnps.ucsd.edu/ProteoSAFe/status.jsp?task=9a7b4f773d734045ae880cb5ebdd34e6
			WT_CF-2_5mg.ml_(23-11-22)_BB8_1_6503.mzXML		
			WT_CF-3_5mg.ml_(23-11-22)_BC7_1_6516.mzXML		
			WT_CF-5_5mg.ml_(23-11-22)_RE4_1_6523.mzXML		
	BOOST	2PRIM	D4_CF-1_5mg.ml_(23-11-22)_BB6_1_6499.mzXML	D4_CF	
			D4_CF-3_5mg.ml_(23-11-22)_BC2_1_6505.mzXML		
			D4_CF-4_5mg.ml_(23-11-22)_BC6_1_6515.mzXML		
			D4_CF-5_5mg.ml_(23-11-22)_RE5_1_6522.mzXML		
311	BOOST_MXP	2PRIM	D4_MXP-1_0.5mg.mzXML	D4_MXP	https://gnps.ucsd.edu/ProteoSAFe/status.jsp?task=c5126a425d894bec8bf94e7219a8c1f1
			D4_MXP-2_0.5mg.mzXML		
			D4_MXP-3_0.5mg.mzXML		
			D4_MXP-5_0.5mg.mzXML		
